# Reduced cognitive function, increased blood-brain-barrier transport and inflammatory responses, and altered brain metabolites in LDLr -/-and C57BL/6 mice fed a western diet

**DOI:** 10.1371/journal.pone.0191909

**Published:** 2018-02-14

**Authors:** Jennifer M. Rutkowsky, Linda L. Lee, Michelle Puchowicz, Mari S. Golub, Douglas E. Befroy, Dennis W. Wilson, Steven Anderson, Gary Cline, Jason Bini, Kamil Borkowski, Trina A. Knotts, John C. Rutledge, on behalf of the Mouse Metabolic Phenotyping Center Imaging Working Group

**Affiliations:** 1 Department of Molecular Biosciences, School of Veterinary Medicine, University of California, Davis, California, United States of America; 2 Division of Cardiovascular Medicine, Department of Internal Medicine, University of California, Davis, California, United States of America; 3 Department of Nutrition, School of Medicine, Case Western Reserve University, Cleveland, Ohio, United States of America; 4 Department of Environmental Toxicology, University of California, Davis, California, United States of America; 5 Magnetic Resonance Research Center, Department of Diagnostic Radiology, Yale University School of Medicine, New Haven, Connecticut, United States of America; 6 Department of Pathology, Microbiology, and Immunology, School of Veterinary Medicine, University of California, Davis, California, United States of America; 7 Department of Physiology and Membrane Biology, University of California, Davis, California, United States of America; 8 Department of Endocrinology, Yale University, New Haven, Connecticut, United States of America; 9 Yale PET Center, Department of Diagnostic Radiology, Yale University, New Haven, Connecticut, United States of America; 10 West Coast Metabolomics Center, Genome Center, University of California, Davis, California, United States of America; Hungarian Academy of Sciences, HUNGARY

## Abstract

Recent work suggests that diet affects brain metabolism thereby impacting cognitive function. Our objective was to determine if a western diet altered brain metabolism, increased blood-brain barrier (BBB) transport and inflammation, and induced cognitive impairment in C57BL/6 (WT) mice and low-density lipoprotein receptor null (LDLr -/-) mice, a model of hyperlipidemia and cognitive decline. We show that a western diet and LDLr -/- moderately influence cognitive processes as assessed by Y-maze and radial arm water maze. Also, western diet significantly increased BBB transport, as well as microvessel factor VIII in LDLr -/- and microglia IBA1 staining in WT, both indicators of activation and neuroinflammation. Interestingly, LDLr -/- mice had a significant increase in ^18^F- fluorodeoxyglucose uptake irrespective of diet and brain ^1^H-magnetic resonance spectroscopy showed increased lactate and lipid moieties. Metabolic assessments of whole mouse brain by GC/MS and LC/MS/MS showed that a western diet altered brain TCA cycle and β-oxidation intermediates, levels of amino acids, and complex lipid levels and elevated proinflammatory lipid mediators. Our study reveals that the western diet has multiple impacts on brain metabolism, physiology, and altered cognitive function that likely manifest via multiple cellular pathways.

## Introduction

Previous studies have shown that elevated blood lipids and a diet high in saturated fats puts individuals at greater risk for dementia and cognitive impairment [1–5]. Moreover, animal studies have shown that a high-fat/high-cholesterol diet not only induces cognitive impairment, but also increases neuroinflammation [6–8]. For instance, low-density lipoprotein receptor null (LDLr -/-) mice are predisposed to elevated blood cholesterol levels and show evidence of cognitive impairment and increased brain inflammation when fed a high fat diet [9–12]. LDLr mediates the endocytosis of cholesterol rich low-density lipoproteins regulating plasma levels of cholesterol. It is prominently expressed in the liver, but also the gastro-intestinal tract, muscle (heart and skeletal) and brain [13]. Genetic knock out of LDLr leads to a twofold elevation in circulating cholesterol and 7-9-fold increase in LDL due to prolonged clearance rate [14]. Our previous work using brain microvascular endothelial cells and astrocytes treated with lipids and lipoproteins showed a complex interaction of multiple cell stress response signaling mechanisms that was not adequately described by a single cell pathway [15–17]. In agreement, a western diet (WD) has been shown to decrease brain capillary expression of tight junction proteins and increase hippocampal blood-brain barrier (BBB) permeability in the rat [18], potentially allowing for additional paracellular movement of blood components including lipids and lipoproteins. Diet has also been shown to activate microglia, resident brain inflammatory cells, and induce inflammation and cellular degeneration [8, 9, 19], each thought to contribute to the progression of cognitive impairment [20].

Other work has linked brain metabolic perturbations with cognitive impairment. For instance, studies using positron emission tomography (PET) to examine regional brain glucose metabolism show that Alzheimer’s disease (AD) and vascular dementia each exhibit a unique pattern of reduced brain glucose uptake [21, 22]. Further, metabolic stress, suggested by the elevation of lactate and glutamate, has been implicated in AD, ischemic stroke, epilepsy, and cognitive impairments [23] and a reduction of N-acetylaspartate accompanied by increases in glutamate & glutamine are correlated with brain injury and cognitive impairment [24–28]. However, the pathways by which major metabolic stressors such as a western diet or hyperlipidemia influence brain metabolite levels and metabolic function are not fully understood.

Therefore, our goal for this project was to better understand the mechanisms of WD-induced cognitive impairment using molecular, cellular, biochemical, physiological, and imaging approaches. Here, we show that in mice, a WD or hyperlipidemia can alter brain glucose uptake and metabolite levels, activate resident inflammatory cells (microglia), increase brain factor VIII vascular expression and the BBB transfer coefficient, and induce moderate cognitive impairments.

We first demonstrated that WD or genetically induced hyperlipidemia moderately impairs cognition as determined by Y and radial arm mazes. Using, Gd-DTPA contrast magnetic resonance imaging (MRI), we determined that a WD increases BBB transfer coefficient (Ki), potentially contributing to cognitive perturbation [18, 29]. Further, indicators of brain inflammation and activation, factor VIII and (ionized calcium binding adaptor molecule 1 (IBA1) protein and prostaglandin-endoperoxide synthase 2 RNA (previously correlated with cognitive disorders), were found to be elevated by WD. As members of the Mouse Metabolic Phenotyping Center Imaging Working Group, we combined the collective and comprehensive expertise of our three universities, to assess how a WD in LDLr-/- and WT mice shifts brain metabolites. Our collaborators at Yale demonstrated an increase in glucose uptake by ^18^F-fluordeoxyglucose (^18^FDG) positron emission tomography (PET) and lactate concentration by ^1^H magnetic resonance spectroscopy (^1^H-MRS) in the brains of live LDLr-/- mice. Our collaborators at Case Western and UC Davis completed a more extensive metabolic analysis to establish that a WD modulates brain fatty acid, TCA cycle intermediates, acyl-CoA’s and complex lipid abundance and elevates proinflammatory lipid mediators. Finally, we review the basic physiological parameters and histopathological effects of diet and genotype revealed by this study that may contribute to the metabolic, inflammatory, and cognitive changes observed.

## Materials and methods

### Mice

All animal studies were approved by the Animal Use and Care Committee at the University of California, Davis and Yale University School of Medicine and were maintained under similar environmental and identical experimental conditions (diet, time on diet, and age at start of diet) at each institution. Animals were euthanized by decapitation or exsanguination under anesthesia (isoflurane or ketamine). Male C57BL/6J (wild type, WT) and low-density lipoprotein receptor knock out mice (LDLr -/-) were imported at ~6 weeks of age from JAX West Laboratory and maintained on a chow diet prior to administering the specialized diet. Mice were housed 2 to a cage that had a transparent divider with small holes to allow visual and olfactory interaction in a temperature controlled room with a 12 h light: 12 h dark cycle. They remained undisturbed except for weekly cage changes and monthly weighs. At eight weeks of age, half the mice in each genotype were fed ad lib either a western (WD; 42% kcal fat, 0.2% total cholesterol, and 34% sucrose by weight) or control diet (CD; 19.2% kcal fat, 0% added total cholesterol, and 12% sucrose by weight) (TD.88137 or TD.08485 respectively, Envigo, Indianapolis, IN) over the course of 12 weeks. Mice were subjected to behavioral analysis prior to sacrifice at 20 weeks of age when non-fasted tissues and plasma were collected. Separate cohorts were used for metabolic profile and immunohistochemistry, as well as cognitive/behavioral analyses (radial arm water maze, Y-maze, and Morris water maze) and MRI analysis of blood-brain barrier transfer coefficient. Plasma samples were analyzed for glucose, insulin, triglyceride, total cholesterol, HDL, and LDL by the UC Davis Mouse Metabolic Phenotyping Center Endocrinology and Metabolism Core according to their standard protocols. For studies of pathology, anesthetized mice were perfusion fixed with 4% paraformaldehyde and sections of brain, liver, heart, kidney, pancreas, skeletal muscle, and lung were embedded in paraffin.

### Cognitive assessment by Y, Morris water, and radial arm water mazes

#### Y-maze

At age 18 weeks, 10 weeks after initiation of feeding, mice were subjected to test of spontaneous alternation in a Y-maze as previously described [30]. Mice, adapted to testing room for 30 min, were placed in the center of the Y-maze and were tracked with an overhead camera for the extent of an 8-min trial. An elevated white plastic Y-maze with three 40 cm arms at 120-degree angles illuminated by red light was used. Entry into each arm, total distance moved, and the amount of time spent resting and active were recorded, and an alternation score was computed as the number of times the three arms were sequentially entered. The % alternation score is the number of alternations divided by the total number of arms entered (n = 8/grp).

#### Morris water maze

The Morris water maze (MWM) was administered at 19 weeks of age over a 5-day period as previously described [31]. The cohort (n = 8/grp) was relocated to a fixed position in the experimental room prior to testing. The first day consisted of a single visible platform trial to ensure that the mice could swim to the platform and climb to the platform surface. For training trials, the platform was hidden below the water surface. Each mouse in the group completed a trial before a new trial was initiated for a total of 4 trials over 4 daily sessions. The water maze was 94 cm in diameter, with a 6 x 6 cm platform whose surface was located 2 cm below the surface of opacified water. Mice were released in one of three locations in non-platform quadrants using a random order that included each location once in the four daily trials. After the mouse reached the platform or 90 s elapsed, the mouse was placed on the platform for 30 s and then returned to a heated cage between trials. A probe trial was conducted on the fifth day, after completion of four daily sessions. The platform was removed from the maze and mice were given one 90 s trial to determine their search strategy for the spatial location of the platform.

#### Radial arm water maze

A separate group of mice was subjected to a different spatial learning and memory test, the radial arm water maze (RAWM) [31]. Mice in the first cohort (n = 5/grp) were tested twice, prior to and after the 12-week experimental diet period to see if there was an early genotype effect and if there was a change with diet (genotype/diet interaction). Mice in the second cohort (n = 5/grp) were tested only after the experimental diet period to evaluate diet genotype interaction to eliminate pre-testing training effects. The test was conducted in a six-arm apparatus (77 cm diameter, 20 cm high, 25 cm long, 14cm wide) placed in a shallow pool of water. The center area was 28 cm in diameter. The escape 6 cm round platform was placed 1 cm below the opacified water surface. The platform was placed at the end of a different designated arm each day of the 9-day test. Each day consisted of 4 consecutive trials (at unique start location) followed by a fifth (retention) trial that was performed 30–40 min following the fourth. Trials began when the mouse was placed in the start arm and ended when the mouse reached the platform or 60 s elapsed. The mouse then remained on the platform for a 30 s inter-trial interval before beginning, then began the next trial.

### MRI measurements of blood-brain barrier (BBB) transfer coefficient (Ki) experimental set-up

BBB transport was assessed using MRI to map regional changes in the longitudinal relaxation time (T_1_) of the brain water signal following an infusion of contrast agent. A minimum of 6 animals from each group were studied and anesthetized with ketamine and xylazine. The left femoral vein was cannulated with PE-10 tubing for administration of saline and gadopentetate dimeglumine (Gd-DTPA—Magnevist; Bayer Healthcare Pharmaceuticals, Wayne, NJ, USA). MRI data were collected using a 7T Bruker Biospec MRS/MRI system (Bruker BioSpin MRI, Inc., Billerica, MA, USA) interfaced with ParaVision 4 (PV4) software (Bruker BioSpin GmbH, Rheinstetten, Germany) with a 32mm radiofrequency (RF) volume coil for transmission and detection. Anesthetized cannulated mice were placed on a PVC animal stage in the prone position. Body temperature was maintained with circulating heated water bed (Gaymar Inc., Orchard Park, NY, USA) while the animal was in the magnet. The mice were placed near the center of the coil such that the isocenter was approximately 1 mm caudal to the bregma.

#### Data acquisition

For each Gd-DTPA infusion, 10 T_1_ maps were obtained consecutively to follow the time course of contrast agent entry into the brain. Each T1 map was acquired using a rapid-acquisition refocused-echo (RARE) sequence with variable T_R_ = 200ms, 531.8ms, 958.6ms, 1557.2ms, 2568.7ms, and 7500ms; effective T_E_ = 30.80ms; RARE-factor = 16. A single slice was taken using 1mm in-plane thickness, 32x32 mm^2^ field of view, 128 x 128-pixel resolution (thus 250 μm x 250 μm x 1 mm voxel size), with a 1.8 min acquisition time per T_1_ map (18 min for 10 consecutive maps). A 0.5 mL/kg aliquot of the contrast agent Magnevist (0.5mmol/mL) was diluted 1:1 with saline and injected i.v. over approximately 10 secs followed by a 50uL saline flush immediately after the acquisition of data for the first T_1_ map in each 10 map series used for Patlak analysis.

#### Data processing and analysis

T_1_ values were calculated using the T_1_ fit function in the Bruker PV4 image sequence analysis tools package. Post-processing image analysis was done using an in-house MATLAB (MATLAB 2014b, MathWorks, Natick, MA) script with Patlak linearized regression mathematical modeling for each pixel. The BBB transfer coefficient, Ki (permeability x surface area/volume - min^-1^), was calculated from the slope of the Patlak plot that best fit an impermeable, uni-directional influx, or bi-directional flux model selected using the F-test with p<0.05 as previously described [32]. Regions-of-interest (ROIs) were manually defined symmetrically about the midline, one from each hemisphere to exclude the median eminence and ventricles. Ki was calculated on a pixel-by-pixel basis and averaged to include both ROIs after pixels with CBF = 0 (see below) were excluded to minimize underestimates of Ki due to flow-limitation of contrast agent delivery [32].

### Perfusion weighted imaging (PWI) analysis of cerebral blood flow (CBF)

#### Data acquisition

CBF data were obtained before and after MRI measurements of BBB transfer coefficient using a Bruker PERF spin-echo sequence with T_R_ = 1022.6ms and T_E_ 12.8ms. Imaging parameters were the same as the RAREVTR but with a 4 min 22 s acquisition time.

#### Data processing and analysis

Post-image acquisition analysis was done using Bruker PV4 image sequence analysis tools package with algebra as described by Williams *et al*. [33] and ROIs were manually defined in the CBF matrix to match the corresponding Ki matrix.

### Assessment of brain factor VIII and IBA1 by immunohistochemistry and general pathology

Analysis of vessel density and microglia activation in brain was determined using factor VIII and IBA1 staining, respectively. Paraffin embedded sections (4 μm) were deparaffinized through xylene to 100% reagent alcohol, and then treated with 0.3% hydrogen peroxide in 100% methanol for 30 min then rehydrated. Antigen retrieval was performed on sections for IBA1 with heat induced epitope retrieval using Target Retrieval Solution, pH 6 (Dako S1699) for 30 min at 95°C, followed by a 20 min cool down and with Proteinase K (Dako S3020) at room temperature for 10 min for factor VIII. Slides were rinsed in deionized water and placed in 0.1M Phosphate Buffered Saline, pH 7.4 (PBS). The antibody diluent and blocking reagent were PBS-Tween 20 (0.02%) and 10% normal horse serum (NHS) in PBS-Tween 20, respectively. Sections were blocked for 20 min followed by the following primary antibodies: factor VIII (Dako A0082, AB_2315602, 1:2000) and IBA1 (Wako 19–19741, AB_839504, 1:600) for 1 h at room temperature. A single step, polymer based HRP (BioCare Medical, RC542H) was applied for 30 min to label rabbit anti-IBA1. A dual step, biotin-avidin based HRP (BioCare Medical, 4+ Detection System GR608) was applied for 10 min to link rabbit anti-factor VIII. Streptavidin-HRP (BioCare Medical HP604) was applied for 10 min to label the biotin link. All labels were visualized with NovaRed for peroxidase (Vector SK-4800), per manufacturer’s instructions. Sections were counterstained in Mayer’s Hematoxylin, air dried and cover slipped.

A coronal section containing cortex, hippocampus, and thalamus at the level of the geniculate bodies and amygdala was used for image analysis. Whole coronal sections were digitally scanned using an Olympus VS110 system. Images then were analyzed with Visiopharm software by manually outlining ROI (cortex, hippocampus, and thalamus) followed by automated detection of relative areas of immunopositivity in each area of interest.

Histopathology was assessed in brain, heart, and liver by hematoxylin and eosin (H&E) staining of four-micron thick paraffin embedded sections (n = at least 5–6 grp). Paraffin was removed with xylene then then sections were rehydrated though a series of decreasing concentrations of ethanol. Sections were then stained with hematoxylin washed and then stained with eosin. Tissue was then dehydrated with ethanol and xylene and a coverslip mounted.

### mRNA expression by quantitative RT-PCR (qRT-PCR)

Total RNA was extracted from powdered mouse brain hemisphere (n = at least 5/group) using TRI Reagent (Sigma-Aldrich, St. Louis, MO) and RNeasy Mini Kit (Qiagen, Valencia, CA) according to manufacturer’s protocol. Up to 5 μg of total RNA from each sample was reverse-transcribed to obtain cDNA in a final volume of 20 μL solution consisting of buffer, random hexamers, DTT, dNTPs and Superscript-III reverse transcriptase (Invitrogen, Carlsbad, CA). qRT-PCR with SYBR Green (Applied Biosystems, Foster City, CA) as fluorescent reporter was used to quantify the expression of selected genes identified by GeneChip analysis. All the gene specific primers (Table 1) were designed with Primer Express 1.0 software (Applied Biosystems) using the gene sequences obtained from Affymetrix Probeset IDs. The reaction was carried out in 384 well optical plates containing 25 ng RNA in each well. Quantitative RT-PCR using the ViiA^™^ 7 Real-Time PCR System (PE Applied Biosystems, Foster City, CA) measured transcript levels. The PCR amplification parameters were: initial denaturation step at 95°C for 10 min followed by 40 cycles, each at 95°C for 15 s (melting) and 60°C for 1 min (annealing and extension). Relative changes in gene expression were determined from real-time quantitative PCR experiments by a ΔΔCT method [34] and was normalized with glyceraldehyde-3-phosphate dehydrogenase (GAPDH). The threshold cycle, Ct, which correlates inversely with the target mRNA levels, was measured as the cycle number at which the SYBR Green emission increases above a preset threshold level. The specific mRNA transcripts were expressed as fold difference from media control in the expression of the specific mRNAs.

**Table 1:** qRT-PCR murine primers. The oligonucleotide sequences for each primer sequence were obtained from Affymetrix database using the probe set IDs and Primer3 software. The primers were custom prepared and used as described in the Methods.

Gene	Forward (5’-3’)	Reverse (5’-3’)
ARG	GAACACGGCAGTGGCTTTAAC	TGCTTAGCTCTGTCTGCTTTGC
ATF3	AGAGCTGAGATTCGCCATCC	GAGGACATCCGATGGCAGAG
C1qA	GCACCCAACGGGAAGGAT	CTTTAAAACCTCGGATACCAGTC
C1qB	TCTGGGAATCCACTGCTGTC	AGACCTCACCCCACTGTGTC
C1qC	CAGCGTCTTCTCTGGTTTCC	TCCTGGAGGAAGAGGTCTGA
CCL2	CTTCTGGGCCTGCTGTTCAC	AGCCAACACGTGGATGCTC
CD11b	GGATCATAGGCGCCCACTT	TCCTTACCCCCACTCAGAGACT
CD16	TTTGGACACCCAGATGTTTCAG	GTCTTCCTTGAGCACCTGGATC
CD32	AATCCTGCCGTTCCTACTGATC	GTGTCACCGTGTCTTCCTTGAG
CD86	TTGTGTGTGTTCTGGAAACGGAG	AACTTAGAGGCTGTGTTGCTGGG
CD206	TCTTTGCCTTTCCCAGTCTCC	TGACACCCAGCGGAATTTC
CXCL2 (MIP2α)	CCAACCACCAGGCTACAGG	GCGTCACACTCAAGCTCTG
DDIT3	AGGGCCAACAGAGGTCACAC	GAATCTGGAGAGGGCT
Factor VIII	GAGGAACCACCGTCAAGCTTCATT	CTGAAGGTGCATAGTCCCAGTCTT
GAPDH	GGATAGGGCCTCTCTTGCTCA	GCAACAGGGTGGTGGACCT
GDF15	GTGTCCCCACCTGTATCGCT	CGTGCTTTGATCTGCGCAT
IBA1	GGATCAACAAGCAATTCCTCGA	CTGAGAAAGTCAGAGTAGCTGA
IGF	ACCCCACCCACAAAACAACA	CGTCCCGGGTCGTTTACAC
IkBα	TCCTGCACTTGGCAATCATC	AGCCAGCTCTCAGAAGTGCC
IL-12a	CCACCCTTGCCCTCCTAAAC	GGCAGCTCCCTCTTGTTGTG
IL-1B	AAGGGCTGCTTCCAAACCTTT	ATACTGCCTGCCTGAAGCTCT
IL- 6	CTCGGCAAACCTAGTGCGTT	GGAATGTCCACAAACTGATATGCT
MAPK8 (JNK)	GCTCCCAGAAAAGCAAGCAG	CATCTTTTGGGGGAGTGCCT
NR4A2	TGCAGGCAGAACCTGAAAGG	CTAAATCCAGGATGCCCCG
PTGS2 (COX2)	CTTAGTTCCGTTTCTCGTGGTCA	AACCCAATCAGCGTTTCTCG
TNFα	GGAACACGTCGTGGGATAATG	GGCAGACTTTGGATGCTTCTT
TREM2	GCCACCTATCCTGGGAACAG	CCAACTCACCACAGATGTACACAC

### PET/CT imaging analysis of brain glucose uptake

At Yale, a cohort of 24 mice were divided into four groups: WT CD (n = 8, mean weight 30.6 ± 3.7 g); WT WD (n = 6, mean weight 34.0 ± 2.9 g); LDLr -/- CD (n = 6, mean weight 29.9 ± 1.5 g); and LDLr -/- WD (n = 4), mean weight (32.9 ± 4.3 g) and were imaged using ^18^FDG PET to assess brain glucose uptake. All mice were injected via tail vein with mean activity of 2.51 ± 3.86 MBq of ^18^FDG and listmode PET data were acquired for 30 min on a preclinical PET/CT scanner (Inveon PET/CT, Siemens Preclinical Systems, Knoxville, TN, USA). All animals were injected while in the scanner under isoflurane anesthesia. All animals had a low-dose CT acquisition after the PET acquisition to provide attenuation correction. All PET acquisitions were reconstructed using the vendor provided reconstruction algorithms (Inveon Acquisition Workplace; Siemens preclinical systems, Knoxville, TN, USA). Images for each acquisition were reconstructed with 3D-OSEM-MAP (OSEM: 2 iterations; 16 subsets) and 18 MAP iterations (β = 0.0023).

#### Image analysis

ROI tracings and region-specific time activity curves (TACs) for the mice were performed using the vendor provided image software viewer (Inveon Research Workplace, Siemens preclinical systems, Knoxville, TN, USA). Due to the incidence in which the radiotracer did not exit the tail vein after injection, we calculated whole body activity at 30 min, using a full body contour ROI minus the tail activity, as our actual injected activity entering the vasculature. Global brain uptake for all mice was determined by drawing a whole brain ROI. Both ROIs were drawn on the co-registered low-dose CT image provided for attenuation correction. To examine differences in ^18^FDG uptake between the four groups, brain ROI TACs were plotted for the entire 30 min acquisition. Based on the initial TACs, brain ROI quantification was also performed on a summed static image from 25–30 min post injection. All quantitative comparisons were performed using the body weight standardized uptake value (SUV) with correction for plasma glucose levels (SUVglu) between groups [35]

### *In vivo* brain metabolomics profiling using ^1^H magnetic resonance spectroscopy (^1^H-MRS)

Concentrations of cerebral metabolites *in vivo* were determined by localized ^1^H MRS on 20-week old male C57BL/6J and LDLr -/- mice after 12 weeks on either a CD or WD. Mice were anesthetized with ~1.5% isoflurane in an air (70%)/ O_2_ (30%) mixture delivered via a fitted nose-cone. The head was kept immobile using a stereotactic restraint with tooth bar and the skull was positioned under a 12mm diameter ^1^H-surface coil. Each mouse was continuously monitored using an MR compatible physiological monitoring system (SA instruments, Stony Brook, NY); body temperature was maintained using a heated water pad and the depth of anesthesia was adjusted as necessary to maintain stable physiological function. MR experiments were performed on a 9.4T/31cm Varian DirectDrive system (Agilent Technologies, Santa Clara, CA), using proprietary and custom-written pulse sequences. Multi-slice gradient echo scout images were obtained to ensure correct positioning and to establish the ROI for spectroscopy. To enhance spectral resolution and sensitivity, magnetic field (B_0_) homogeneity was optimized over a 3x3x3mm volume using a custom-written, adiabatic Fastmap sequence; typical ^1^H_2_O linewidths within the region of interest were 15–17 Hz. Localized, water-suppressed, ^1^H MR spectra were obtained from an 18.75 μL volume (2.5x2.5x3mm) using a LASER (Localization by Adiabatic Selective Refocusing) sequence [36] (1ms AHP excitation pulse, 2ms AFP refocusing pulses, sw = 6kHz, 4k points, T_R_ = 3s, T_E_ = 27.3ms) preceded by chemical shift selective (CHESS) water suppression (6x 10ms AFP pulses). Spectra were acquired from the cortex of each hemisphere over 256 transients; total acquisition time for each hemisphere was ~13min.

Spectral processing was performed using MATLAB (Mathworks Inc, Natick, MA) software developed in house at the Yale Magnetic Resonance Research Center by Dr. Robin deGraaf and Dr. Douglas Befroy. The relative concentrations of cerebral metabolites were estimated using LCModel 6.3 [37]. Spectral fitting was performed using simulated spectra of a basis set of 17 metabolites (Alanine, Aspartate, β-Hydroxybutyrate, Choline, Creatine, γ-aminobutyric acid (GABA), α-Glucose, β-Glucose, Glutamate, Glutamine, Glutathione, Lactate, MyoInositol, NAA, PhosphoCholine, PhosphoCreatine, Taurine). The contribution of macromolecules to the *in vivo* spectrum was fitted using an *in vivo* macromolecule reference spectrum (composite of 4 regions) acquired with the metabolite peaks suppressed using a double-inversion module in the LASER sequence; lipid components were fitted using the standard resonances in the LCModel package. Metabolite content is expressed relative to the total creatine (Cr+PCr) content; estimates for individual metabolites with Craemer-Rao lower bounds (CRLB) >20% were discarded from the group analysis.

### Brain metabolite analyses

Targeted metabolic profiling was conducted at Case Western on whole homogenate brain tissues collected from 20-week-old mice (C57BL/6 and LDLr -/-) fed either CD or WD for 12 weeks. Tricarboxylic acid (TCA) cycle intermediates, total (free + bound) lipids and cholesterol, acyl-CoA’s and related amino acids, such as glutamine, glutamate, and aspartate, as well as ketone body (beta-hyroxybutyrate; BHB) and lactate were quantitatively assayed using sensitive gas chromatography (GC/MS) [38–41] and liquid chromatography (LC/MS/MS) mass spectrometry [42–44] analyses. Each of the selected intermediates was quantified against reference standards to yield absolute concentrations (μmol/g wet weight tissue). Following decapitation under isoflurane twilight anesthesia, the brains from each mouse were rapidly collected and quickly frozen under liquid nitrogen [41, 43]. Metabolic profiles were conducted on a single mouse brain. Brain tissues (~200 mg, cortical-subcortical/mouse) were homogenized in a methanol-5% acetic acid (in milli-Q water) buffer solution (3 ml) following additions of reference standards. Before centrifugation an aliquot (0.2 ml) of homogenate was reserved for fatty acid and cholesterol assays and the remaining homogenate then was centrifuged and the supernatant fraction (extract) was collected and divided into three additional aliquots. One aliquot (2.2 ml) from the extract was used for acyl-CoA analysis (LC/MS/MS), and the other aliquots were used for GC/MS analysis.

#### Analysis of TCA and amino acid metabolite

For measurements of TCA and amino acid metabolites: an aliquot of extract (0.2 ml) was dried by nitrogen gas for 1–2 hr. and derivatized using MTBSTFA + 1% TBDMCS reagent (N-methyl-N-(tert-butyldimethylsilyl) trifluoroacetamide + 1% tert-butyldimetheylchlorosilane, Regis Technologies, Inc. Morton Grove, IL, USA) and reacted at 70°C for 30 min [39–41]. Each of the derivatized products were analyzed as trimethylsilyl derivatives on an Agilent 5973N-MSD equipped with an Agilent 6890 GC system (GC-MS) coupled to a DB-17MS capillary column (30m x 0.25mm x 0.25 μm) and operated in electron impact ionization (EI) sim mode during increasing oven temperature gradient. The corresponding ions monitored for each metabolite were as follows: BHB (m/z = 159), succinate (m/z = 289), fumarate (m/z = 287), (malate m/z = 419), citrate (m/z = 459). Other intermediates and amino acids, including 3-hydroxyglutarate (m/z = 433), aspartate (m/z = 418), glutamate (m/z = 432), glutamine (m/z = 431) and GABA (m/z = 274) were also measured. The analysis of lactate involved using 0.05 ml of extract and the GC/MS system as describe above, but slightly modified methods. Lactate (m/z = 131) was analyzed as a PFBBR derivative and operated in chemical ionization mode (CI, ammonia gas) [42].

Fatty acid and cholesterol concentrations were analyzed from the aliquot reserved from the homogenate and then dried under nitrogen gas. The individual lipids were analyzed as trimethylsilyl derivatives using the GC/MS system as described above (EI sim mode) [38, 45]. The corresponding ions monitored for each of the lipids were as follows: C:14 (m/z = 285), C16:0 (m/z = 313), C17:0 internal standard (m/z = 327), C:16:1 (m/z = 311), C:18 (m/z = 341), C18:1 (m/z = 339), C18:2 (m/z = 337), C:20 (m/z = 369), C:20:1 (m/z = 367), C:22 (m/z = 397) and cholesterol (m/z = 368).

#### Acyl-CoA profiles analysis

Acyl-CoA profiles were assayed from the remaining extract (~2.2 ml) [41–44]; extract was loaded onto a Supelco solid-phase extraction cartridge ([2-(pyridyl)-ethyl functionalized silica gel]) pretreated with methanol. The cartridge was washed with a buffer containing methanol with 2% acetic acid, followed by elution of acyl-CoAs using buffers containing ammonium formate and/or methanol. The eluent was evaporated under nitrogen gas, and applied to LC/MS/MS. The LC was coupled with an API4000 Qtrap MS (Applied Biosystems, Foster City, CA, USA) operated under positive ionization mode, and the Q1/Q3 components were monitored for each acyl-CoA species (C2:0 –C20:4 CoA).

#### Complex lipids

Whole brain metabolic analysis of complex lipids was assessed by the West Coast Metabolomic Center at UC Davis by charged-surface hybrid column electrospray ionization quadrupole time of flight tandem mass spectrometry (CSH-ESI QTOF MS/MS) in both positive and negative modes using methods described previously [46–49]. Samples were extracted using the Matyash protocol using methyl tert-butyl ether (MTBE) [50]. 4 mg of pulverized brain sample was mixed with 225 μL of ice-cold degassed MeOH and vortexed for 20s, 750 μL of ice-cold degassed MTBE was then added followed by vortexing for 20s and homogenize for 30s. MilliQ water was added (188 μL) followed by vortexing (20 s) and centrifugation (2 min; 14,000 g). The resulting upper phase is then transferred (350 μL) to a separate tube, dried, and reconstituted with 65 μL MeOH:toluene+CUDA (9:1, v/v). Aliquots of 30 μL were transferred to two separate vials with micro-inserts for UHPLC-QTOF-MS analysis. Samples (3 μL) were injected at 65°C and separated using a Waters Acquity UPLC CSH C18 column (100mm× 2.1 mm) with a particle size of 1.9 μm and a flow rate of 0.6 mL/min. Mass spectrometry was conducted for positively charged ions (phosphatidylcholine (PC), lysoPC, PE, and PS) with an Agilent 6530 QTOF MS (resolution: 10,000) and for negatively charged ions (free fatty acids and phosphatidylinositols) with an Agilent 6550 QTOF MS (resolution: 20,000). Both mass spectrometers operated at full scan range m/z 65–1,700. Peak identification was processed in MassHunter Qual (Agilent) using the MS/MS information and Fiehn laboratory LipidBlast spectral library [51] and then imported to Mass Profiler Professional for peak alignment. Results are provided as quantifier ion peak heights and normalized to the sum of all peak heights for all identified metabolites for each sample. In-depth details of the protocol can be found through the Metabolomics Workbench under protocol number 163 (http://www.metabolomicsworkbench.org/protocols/protocoldetails.php?file_id=163).

#### Non-esterified oxylipins and endocannabinoids analysis

Oxylipins, endocannabinoids, and fatty acids were isolated using methanol:ethyl acetate liquid-liquid extraction protocol from ~25 mg of the mouse brain. Procedure shortly: ~25 mg mouse brain was homogenized using Geno rider 2000 sample homogenizer (SPEX Sample Prep; Metuchen, NJ) together with 5 μL BHT/EDTA (1:1 MeOH:water), 5 μL of 1 μM deuterated surrogates in methanol and 95 μL of methanol. Further, samples were mixed with 0.5 mL of deionized water and 1 mL of ethyl acetate and subsequently vortexed and centrifuged (10 min at 15,000 rcf). The organic phase was retreated and dried by applying a vacuum at 15 Hg for 10 min. Samples were reconstituted in 100μl of 1-cyclohexyl ureido, 3-dodecanoic acid (CUDA) and 1-Phenyl 3-Hexadecanoic Acid Urea (PHAU) at 100nM in 1:1 methanol:acetonitrile. Residues within extracts were separated on a 2.1 x 150mm 0.17μm BEH column (Waters) and detected by electrospray ionization with multi reaction monitoring on a API 6500 QTRAP (Sciex; Redwood City, CA) and quantified against 7–9 point calibration curves of authentic standards using modifications of previously reported methods [52, 53].

### Statistics

Values are reported as mean ± SEM unless otherwise noted. Statistical calculations were done with the Sigma Stat software (Systat Software Inc., San Jose, CA), JMP (SAS institute Inc, Cary, NC, USA), and GraphPad Prism software (GraphPad Software Inc., La Jolla, CA). Two-way ANOVA tables were generated as previously described [54]. Grubb’s test was used to exclude outliers and samples not normally distributed were transformed using $\frac{1}{x},\sqrt{x}$, or log *x*. Student t-tests, or one-way or two-way analysis of variance (ANOVA) followed by Tukey or Student-Newman-Keuls (SNK) post-hoc tests were used to test for differences between groups as indicated in the figure legends and statistical differences assigned at *p*≤0.05. Non-esterified oxylipins and endocannabinoid data were Log transformed and auto-scaled before multivariate analysis, as previously reported [55]. Statistical analyses including; partial least squares analysis, variable hierarchical clustering and repeated measures ANOVA were performed in JMP-Pro v 12.2. Repeated measures ANOVA was performed on variable clusters with a random subject effect, whereas variable cluster members, diet and genotype were used as fixed effects. The model tested for the diet, genotype, and the diet x genotype interaction effect.

## Results

### Western diet leads to poorer Y-maze alternation and better Morris maze retention while LDLr -/- mice were more active and showed impaired learning in the radial arm maze

**Fig 1:**
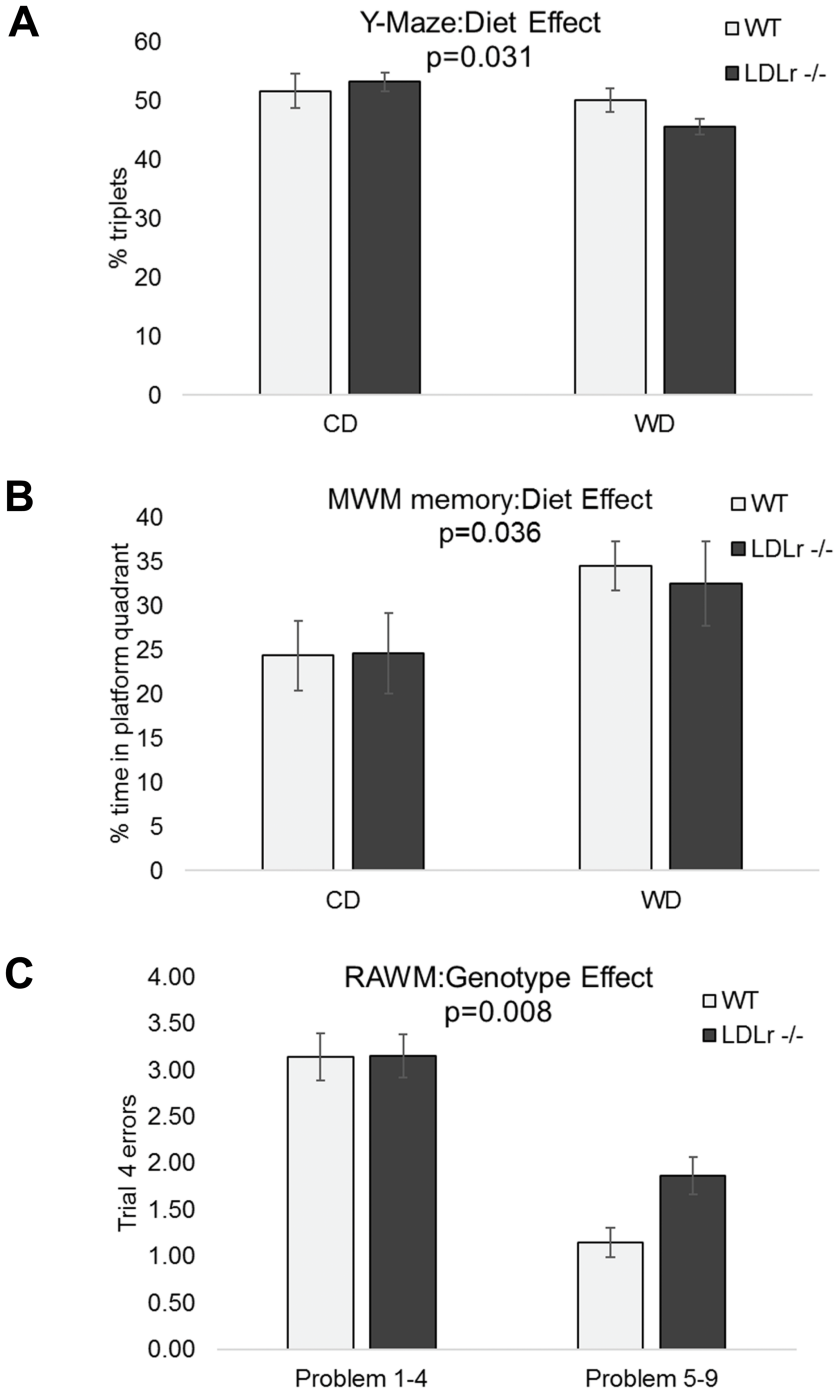
Diet and genotype effects on cognitive endpoints from three different behavioral tests. The performance of C57BL/6 (WT) or LDLr-/- mice in the Y-maze alternation was determined after they were fed a control (CD) or western (WD) diet (A). The mean percentage of triplicates (number of alternations, number of times 3 adjacent arms, divided by the total number of arms entered) is shown (n = 8/grp). These same animals were also exposed to the Morris Water Maze (MWM, B) where % time spent in the platform quadrant during the probe trial is shown. In separate but identically treated groups of WT and LDLr -/- animals, mice were exposed to the Radial Arm Water Maze (RAWM, C) and the number of trial 4 errors in day’s 1–4 vs 5–9 of testing is shown (n = at least 9/grp). Values are mean ±SEM and analyzed by two-way ANOVA with Tukey’s multiple comparisons post hoc test.

To assess the cognitive effects of LDLr -/- and/or WD mice were tested with either both the Y-maze and Morris water maze (MWM), or the radial arm water maze (RAWM) at the age of 20 weeks (12 weeks on defined diet). In addition to cognitive performance as reflected in correct response, performance variables (like speed and thigmotaxis) were analyzed for each of the maze tests. In the Y-maze, LDLr -/- mice were consistently more active than C57BL/6 (WT) with an increased number of total arm entries (63.9±2 vs 54.3±2), total distance travelled (4103.7±104 vs 3538.2±102.5), and decreased % resting time (10.3±.06 vs 14.8±1.0). There was no effect of diet on performance endpoints; however, diet significantly affected the cognitive endpoint % alternation triplets regardless of genotype (*p*≤0.05). Poorer performance (fewer alternation triplets) was seen in the WD-fed group (Fig 1A). There was also a trend (*p* = 0.06) toward an interaction between genotype and diet, with the diet effect more prominent in the LDLr -/- subgroup.

The MWM included visible, training, and probe trials. No effects of diet or genotype were seen for the ability of the mice to find a visible platform trial and this performance was not associated with body weight at the end of behavioral testing (20 weeks of age). With the training trials, there were no effects of diet or genotype seen for performance endpoints (floating, thigmotaxis, and swimming speed). Similarly, no effects were seen on the cognitive measures of learning, the decrease in distance and time required to reach the platform between the first and last day of training. As a group (WT and LDLr-/-), the mice decreased distance to platform by 43±5% and time to platform by 30±7% during training. While there were no effects of diet or genotype on performance endpoints (swim speed, float time, or time in “wall zone”) in the probe trial, a diet effect (*p*≤0.05) was seen for the cognitive endpoint % time in the platform quadrant (Fig 1B). This finding was supported by a trend toward a diet effect (*p* = 0.09) for mean distance to target, or the ability of the mice to maintain a search pattern close to the platform. In both cases, the WD group showed greater time in platform quadrant and reduced distance to quadrant compared to the CD fed group.

Since the MWM and Y-maze were run on the same animals, we determined whether there was an association between the results of these tests by principal components analysis (PCA). We found no correlation between the main cognitive endpoints of the MWM and Y-maze, % platform quadrant time in the probe trial and % alternation triplets, (r = -0.03). This indicated that the two endpoints were measuring distinct cognitive processes with no interdependence in determining behavior for these endpoints. Body mass at the time of testing, cognitive, and performance endpoints were entered into PCA to determine the associations among these endpoints. Analysis indicated that performance variables were not strongly associated with cognitive variables in either task.

To assess spatial learning and memory with a more complex test of learning, two additional cohorts of mice were tested by RAWM either pre- and post-12-week experimental diet intervention (twice), or only after the experimental diet intervention. We found no genotype effects on performance endpoints (float time or latency to platform) in mice tested pre-experimental diet. In addition, no genotype or diet effects were seen on performance endpoints in mice only post-tested after completion of the dietary exposure. We found that the initial pre-diet testing substantially improved the learning of the mice at the post-experimental diet test. Mice who had been tested previously averaged 1.8±0.2 errors during the first 4 RAWM sessions as compared to 3.4±0.2 errors for mice who had not been tested previously. Therefore only the first testing experience (cohort 1 = pre-diet, cohort 2 = post diet) was used to assess genotype effects. The LDLr -/- mice made the same number of errors as WT on the early problems (day 1–4), but made more on the later problems (days 5–9) demonstrating lack of improvement as seen in Fig 1C. Since both WD and LDLr-/- induce altered cognitive function, subsequent analysis of BBB transport, glucose uptake, neuroinflammation, and brain metabolites were assessed in both models as well as potential interaction.

### Western diet feeding increases blood-brain barrier transport

**Fig 2:**
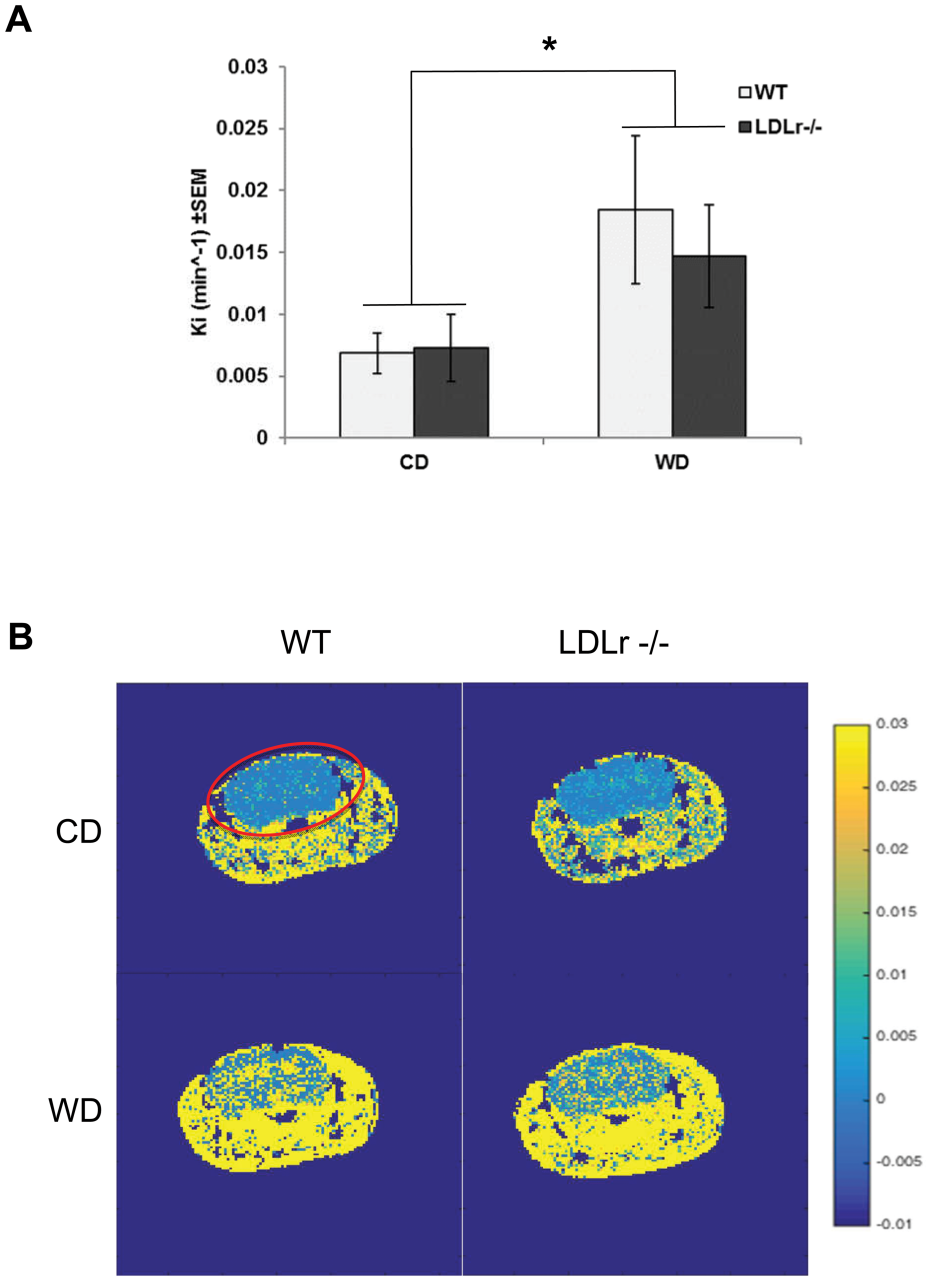
Western diet increases BBB transfer coefficient (Ki). Wild type (WT) and LDLr -/- animals on either a control (CD) or western diet (WD) are plotted against their mean Ki (min^-1^) ± SEM (A). Representative images of the Ki maps of WT CD, LDLr -/- CD, WT WD and LDLr -/- WD are shown (B) where brain is circled in red. Two-way ANOVA showed a significant effect of diet on the BBB transfer coefficient Ki where WD increased Ki when WT and LDLr-/- data were pooled denoted by *, *p*<0.05 (n = at least 6/grp).

WT and LDLr -/- mice fed a CD or WD were subjected to MRI analysis to determine if diet and/or the hyperlipidemic genotype altered BBB transport, indicated by a change in transfer coefficient (Ki = permeability x surface area/volume). Two-way ANOVA showed a significant effect of diet on the BBB transfer coefficient Ki where WD increased Ki when WT and LDLr-/- data were pooled (*p*≤0.05, Fig 2A). There was no significant difference between the genotypes nor was there a significant interaction between genotype and diet (*p* = 0.653 and 0.578, respectively). Increased Ki can be seen in the brain (circled in red) as a shift to yellow in the representative images of Ki maps for each group (Fig 2B). These shifts in Ki were not associated with measurable differences in blood flow as perfusion weighted imaging of cerebral CBF to the brain was essentially normal at 1.7±0.025 mL/100gm/sec and significant differences between mean CBF measured for phenotypes and genotypes were not found (data in supplement). Further, post-imaging venous blood samples were drawn from the vena cava and there were no significant differences in pH, pCO2, pO2 levels between the groups (data in supplement).

### Altered vessel density and microglia activation in western diet fed mice

**Fig 3:**
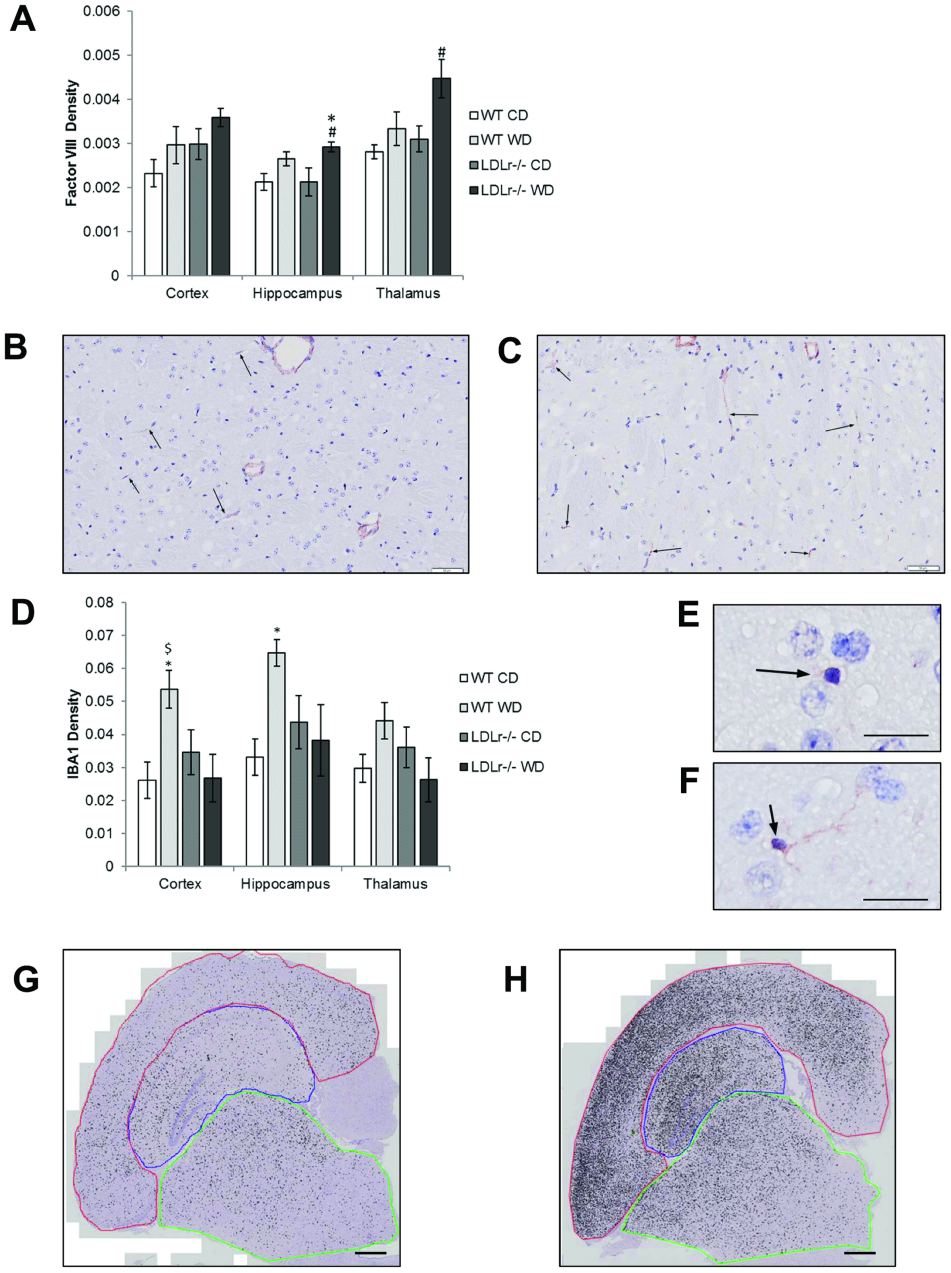
Factor VIII immunostaining density increases in hippocampus and thalamus of LDLr -/- while IBA1 increases in the cortex and hippocampus of C57BL/6 (WT) fed a western diet. Area density of factor VIII and IBA1 immunostaining in coronal section of C57BL/6 (WT) or LDLr -/- mice fed control (CD) or western diet (WD) as determined by image analysis (A and D). Representative images comparing factor VIII immunostaining in thalamus of LDLr -/- mice fed CD (B) or WD (C) (scale bar = 50μm). WD fed animals had more granules of factor VIII positive material in capillaries (arrows). Microglia in control fed mice (E) have most IBA-1 positivity surrounding the nucleus while those from western diet (F) fed animals have denser staining that extends through elongate strands of cytoplasm (scale bar = 20μm). Representative images comparing IBA1 immunostaining in cortex (red), hippocampus (blue) and thalamus (green) of WT mice fed CD (G) or WD (H) (scale bar = ~0.5mm). Statistical differences from WT (*) or LDLr -/- (#) CD fed mice or LDLr-/-WD fed ($), as determined by ANOVA with Tukey’s multiple comparisons post hoc test *p*<0.05, n = at least 5/grp).

To better understand the influence of a WD on brain vascularization, an immunohistological analysis of vessel density was determined by factor VIII staining. We found a significant increase in the relative surface area stained with factor VIII in the hippocampus of LDLr -/- WD-fed mice relative to both control WT or LDLr -/- mice fed a CD, and in the thalamus compared to WT CD-fed mice (Fig 3A). Subjective evaluation suggests that this represents an increase in factor VIII expression in individual endothelial cells in larger vessels as well as expression in capillary endothelium that has a paucity of staining in other groups (Fig 3B and 3C). Subjective histopathologic analysis found no overt differences between genotype and diet groups in brain sections examined nor planimetry measurements of overall surface area for the mid temporal and occipital cortex.

To determine the extent and location of microglia activation in the brains of WD-fed and/or LDLr -/- mice, brain sections were stained for IBA1, which labels microglia and is increased in activated microglia. There was a significant increase in IBA1 staining in WT mice fed a WD compared with mice fed the CD in both the cortex and hippocampus (Fig 3D–3H). In addition, microglial cells had markedly more prominent processes (Fig 3E and 3F). The density of IBA1 staining in both CD- and WD-fed LDLr -/- mice were no different from WT CD fed mice. Similar trends in the thalamus were not statistically significant, in part due to variability in the extent to which the cerebral peduncles were included in the section evaluated. Subjective observation clearly demonstrated that the cerebral peduncles contained a significantly greater density of IBA1 positive cells regardless of diet of genotype group.

### Diet or genotype effects on inflammatory gene expression

Since we found a significant increase in IBA1 staining in WT mice fed a WD, we attempted to determine the type of inflammatory response that was occurring. For this, whole brain expression of genes known to be associated with an M1 or M2 microglia activation (CD16, CD32 & CD86 vs CD 206 & arginase 1) [56, 57], neurodegeneration (IL-1B and IL-6), neuroprotection (CX3CR1, TREM2, IGF1, GDF15, IL10) vascular inflammation (factor VIII and TNFα) or additional stress and inflammatory pathways (ATF3, CHOP, JNK, NFκβ) were assessed. While many of these showed no significant difference by genotype (WT vs LDLr -/-), diet (CD vs WD), or diet x genotype interaction, we found a significant decrease in ATF3, CD206, CD32, and IL-6 in LDLr -/- mice (when compared to WT) and a significant decrease in CD86 and a trend for a decrease in IBA1 with a WD (Table 2). In addition, prostaglandin-endoperoxide synthase 2 (PTGS2, also known as COX2) was significantly increased with a WD.

**Table 2:** Shift in gene expression with western diet and/or Ldlr -/-. C57BL/6 (WT) or LDLr-/- mice were fed a control (CD) or western (WD) diet for 12 weeks and their brain gene expression was measured. Values are fold of WT on a CD ±SEM. Means in a row without a common subscript letter differ (p≤ 0.05) as analyzed by two-way ANOVA and Tukey test. GxD = Genotype x Diet interaction effect (n = at least 5/grp).

Variable	WT		LDLr -/-		*P*-value		
	CD *(n = 6)*	WD *(n = 5)*	CD *(n = 6)*	WD *(n = 6)*	Genotype	Diet	G×D^1^
ARG	1.06 ± 0.153	1.21 ± 0.22	0.933 ± 0.131	0.85 ± 0.0737	0.127	0.844	0.434
ATF3	1.02 ± 0.0988	0.956 ± 0.0453	0.863 ± 0.0286	0.833 ± 0.0205	0.023	0.426	0.763
C1qA	1.01 ± 0.0514	1.03 ± 0.104	0.833 ± 0.103	0.989 ± 0.0667	0.256	0.306	0.42
C1qB	1 ± 0.0109	0.95 ± 0.091	0.921 ± 0.0678	0.943 ± 0.065	0.494	0.828	0.584
C1qC	1 ± 0.0308	1.01 ± 0.088	1.04 ± 0.0332	1.08 ± 0.0522	0.335	0.688	0.836
CCL2	1.03 ± 0.0995	1.04 ± 0.0434	0.929 ± 0.0862	0.847 ± 0.0471	0.075	0.618	0.561
CD11b	1.01 ± 0.0616	1.06 ± 0.0537	1.14 ± 0.0801	1.07 ± 0.0515	0.287	0.839	0.367
CD16	1 ± 0.0441	0.957 ± 0.0346	1.07 ± 0.135	0.979 ± 0.0689	0.629	0.411	0.8
CD32	1.02 ± 0.0877^a^	1.01 ± 0.0465^ab^	0.782 ± 0.0369^bc^	0.753 ± 0.0406^c^	<0.001	0.709	0.894
CD86	1.04 ± 0.118	0.957 ± 0.0637	1.14 ± 0.0623	0.869 ± 0.0715	0.973	0.042	0.273
CD206	1.07 ± 0.162	1.02 ± 0.0717	0.83 ± 0.0468	0.783 ± 0.0311	0.02	0.595	0.976
CXCL2	1.01 ± 0.0513	1.48 ± 0.41	1.18 ± 0.0795	0.995 ± 0.0866	0.473	0.473	0.114
DDIT3	1.01 ± 0.0507	1.01 ± 0.0381	1.12 ± 0.106	0.964 ± 0.0592	0.627	0.269	0.298
Factor VIII	1.04 ± 0.124	1.1 ± 0.0806	1.11 ± 0.0815	1.03 ± 0.0472	0.992	0.927	0.471
GDF15	1 ± 0.0387	1.07 ± 0.066	1.14 ± 0.126	1.14 ± 0.11	0.258	0.738	0.762
IBA1	1.01 ± 0.0487	0.912 ± 0.0291	1.14 ± 0.132	0.921 ± 0.0348	0.394	0.053	0.431
IGF	1.08 ± 0.186	1.36 ± 0.207	0.955 ± 0.0489	0.988 ± 0.179	0.169	0.366	0.457
IkBα	1.04 ± 0.136	1.15 ± 0.064	1.11 ± 0.112	1.32 ± 0.204	0.395	0.276	0.75
IL-12a	1.4 ± 0.616	0.851 ± 0.119	0.789 ± 0.068	1.23 ± 0.213	0.697	0.938	0.173
IL-1B	1.03 ± 0.103	0.971 ± 0.129	0.821 ± 0.123	0.711 ± 0.114	0.058	0.481	0.822
IL- 6	1.03 ± 0.112^a^	0.821 ± 0.191^ab^	0.536 ± 0.061^b^	0.572 ± 0.0925^b^	0.004	0.494	0.305
MAPK 8	1.02 ± 0.101	1.29 ± 0.19	1.04 ± 0.0821	0.899 ± 0.0603	0.125	0.628	0.081
NR4A2	1.01 ± 0.0784	0.994 ± 0.0758	0.902 ± 0.046	0.988 ± 0.0504	0.355	0.581	0.408
PTGS2	1.12 ± 0.231	1.3 ± 0.0868	0.942 ± 0.174	1.49 ± 0.172	0.952	0.048	0.322
TNFα	1.01 ± 0.0712	1.07 ± 0.0447	0.989 ± 0.0357	0.948 ± 0.0354	0.178	0.933	0.352
TREM2	1.02 ± 0.0839	1.04 ± 0.108	1.3 ± 0.141	1.05 ± 0.109	0.214	0.289	0.231

### LDLr -/- mice show altered brain glucose metabolism

**Fig 4:**
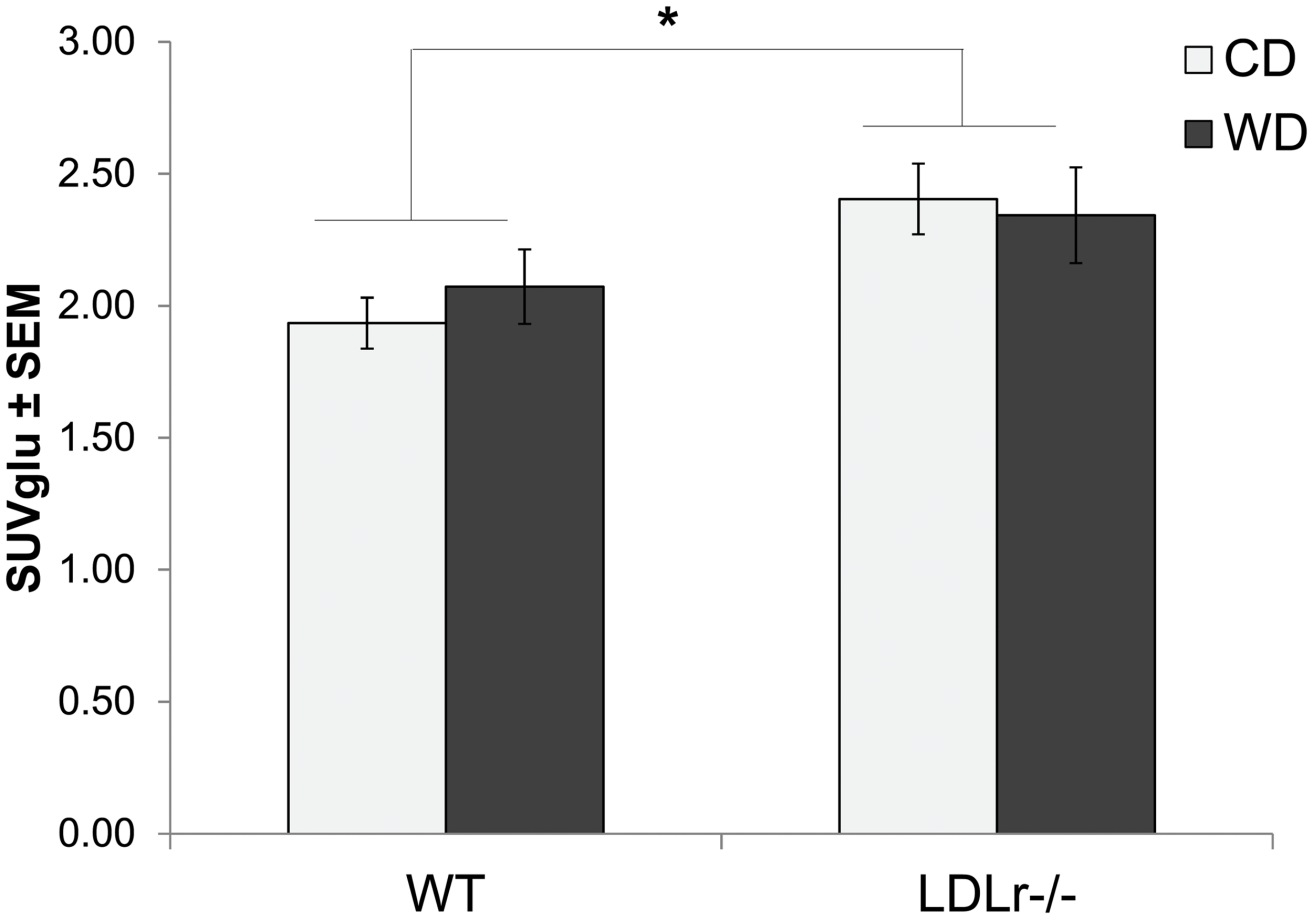
Genotype but not diet alters brain glucose uptake. ^18^FDG uptake measured by mean standardized uptake value (SUVglu) corrected for plasma glucose from a summed static image from 20–30 min post injection of C57BL/6 (WT) or LDLr -/- mice fed control (CD) or western diet (WD). Statistical differences by two-way ANOVA between WT and LDLr -/- mice (*) *p*<0.05, n = at least 4/grp.

To determine the effects on brain glucose uptake, C57BL/6 (WT) and LDLr -/- mice fed either a WD or CD underwent ^18^FDG PET/CT imaging. ^18^FDG SUVglu values for brain ROIs in each respective group were calculated from summed PET images from 25–30 min post-injection (Fig 4) where LDLr -/- showed a significant increase in glucose uptake compared to WT mice (2.37 ± 0.10 and 2.00 ± 0.09, *p*≤0.05).

### Increase of lactate in LDLr-/- mice and lipid moieties by proton magnetic resonance spectroscopy (^1^H-MRS) in LDLr -/- mice fed a WD

**Fig 5:**
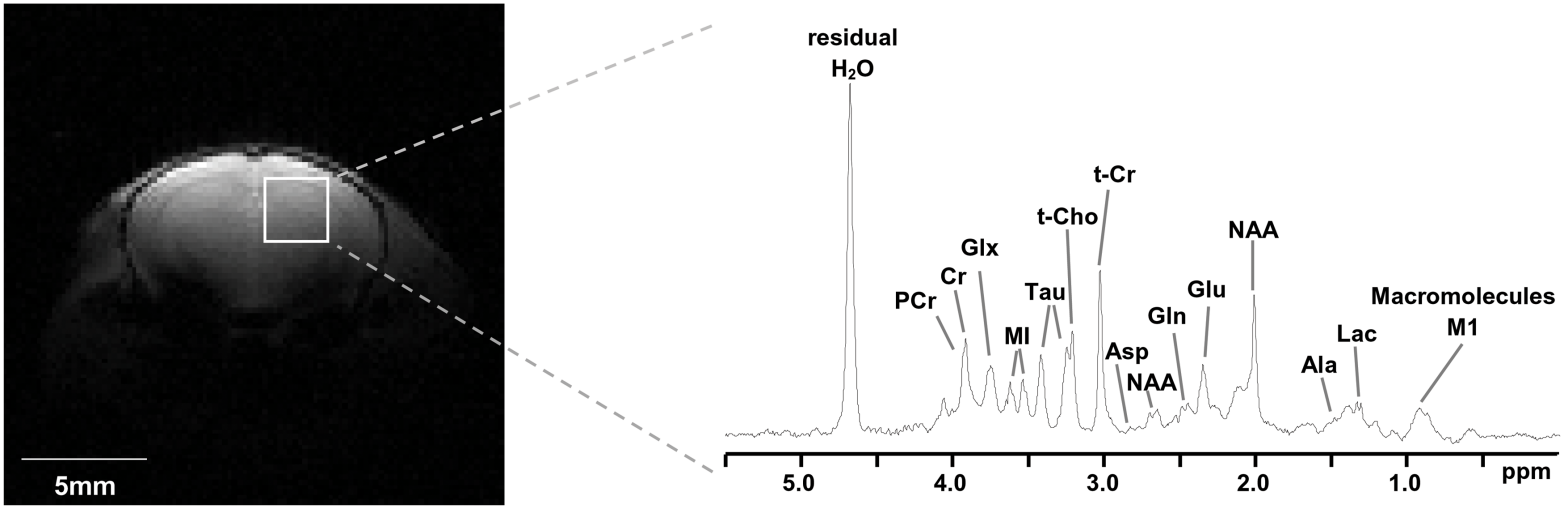
In vivo brain metabolite content determined by ^1^H-MRS. Localized ^1^H spectra were obtained from an 18.75 μl volume (white box) positioned within the left or right hemisphere of the brain. Metabolite content was determined by spectral fitting with LC model using a basis set of 17 metabolites plus components due to macromolecules and intracellular lipids.

To measure shifts in metabolites *in vivo*, ^1^H-MRS were analyzed using spectral fitting to estimate the content of 17 discrete metabolites plus lipid and macromolecules within the cortex (representative image and spectra shown in Fig 5). Lactate concentrations were significantly increased in the LDLr -/- mice compared to C57BL/6 (2.06±0.14 and 1.47±0.13, *p*≤0.05). In addition, LDLr -/- mice fed a WD had higher lipid concentrations (lipid 13a, lipid 9 and lipid 13a+13b) compared to LDLr -/- on CD or to WT on either diet (Table 3). While not significant, there was a trend (*p* = 0.084) for glutamine to decrease in the cortex of LDLr -/- when compared to control mice (2.92±0.14 and 3.70 ±0.41, respectively).

**Table 3:** Shift in brain metabolites assessed by ^1^H-MRS by western diet and/or LDLr -/-. C57BL/6 (WT) or LDLr-/ mice were fed a control (CD) or western (WD) diet for 12 weeks and their brain fatty acid and TCA cycle intermediates were measured. Values are mean of abundance relative to Cr+PCr ±SEM. Means in a row without a common subscript letter differ (p≤ 0.05) as analyzed by two-way ANOVA and Tukey test. GxD = Genotype x Diet interaction effect (*p*≤ 0.05, n = at least 7/grp).

Metabolite	WT		LDLr -/-		*P*-value		
	CD *(n = 7)*	WD *(n = 8)*	CD *(n = 8)*	WD *(n = 7)*	Genotype	Diet	G×D^1^
Alanine	1.2 ± 0.303	1.21 ± 0.143	1.12 ± 0.195	1.31 ± 0.319	0.951	0.691	0.727
Aspartate	3.2 ± 0.254	3.49 ± 0.375	3.6 ± 0.238	3.69 ± 0.327	0.387	0.566	0.765
Choline	0.598 ± 0.0718	0.726 ± 0.0676	0.663 ± 0.0767	0.566 ± 0.0826	0.517	0.836	0.146
Creatinine	5.19 ± 0.122	5.02 ± 0.107	5.18 ± 0.132	4.96 ± 0.164	0.872	0.152	0.88
GABA	1.42 ± 0.121	1.66 ± 0.0956	1.64 ± 0.179	1.5 ± 0.0827	0.798	0.692	0.155
αGlucose	1.65 ± 0.258	1.54 ± 0.148	1.39 ± 0.0925	1.46 ± 0.11	0.3	0.879	0.584
βGlucose	2.17 ± 0.316	2.1 ± 0.214	1.84 ± 0.147	1.94 ± 0.241	0.299	0.954	0.741
Glutamate	7.95 ± 0.282	8.17 ± 0.106	8.16 ± 0.315	8.36 ± 0.38	0.514	0.463	0.957
Glutamine	3.79 ± 0.639	3.63 ± 0.557	3.25 ± 0.174	2.56 ± 0.102	0.084	0.34	0.549
Glutathione	1.73 ± 0.121	1.65 ± 0.106	1.69 ± 0.0912	1.72 ± 0.0646	0.873	0.774	0.546
Lactate	1.79 ± 0.183^ab^	1.35 ± 0.163^b^	2.15 ± 0.191^a^	1.92 ± 0.227^ab^	0.018	0.186	0.666
MyoInositol	5.14 ± 0.295	5.21 ± 0.3	5.19 ± 0.0985	5.41 ± 0.218	0.633	0.55	0.756
NAA	6.06 ± 0.16	5.97 ± 0.129	5.94 ± 0.214	5.83 ± 0.113	0.47	0.525	0.963
PhosphoCholine	0.944 ± 0.0636	0.855 ± 0.0592	0.916 ± 0.0839	0.967 ± 0.0726	0.546	0.794	0.332
PhosphoCreatine	2.81 ± 0.122	2.98 ± 0.107	2.82 ± 0.132	3.04 ± 0.164	0.872	0.152	0.88
Taurine	6.73 ± 0.472	6.47 ± 0.221	7.01 ± 0.281	6.79 ± 0.405	0.367	0.496	0.957
MM C57	0.0667 ± 0.00303	0.0716 ± 0.00236	0.0695 ± 0.0041	0.0683 ± 0.00197	0.896	0.549	0.324
Cr+PCr	8 ± 0	8 ± 0	8 ± 0	8 ± 0	0.324	0.292	0.292
Glu+Gln	11.7 ± 0.643	11.8 ± 0.53	11.4 ± 0.22	10.9 ± 0.421	0.22	0.658	0.558
Lip13a	5.04 ± 0.353^ab^	4.72 ± 0.584^ab^	4.11 ± 0.389^b^	9.04 ± 2.31^a^	0.202	0.054	0.038
Lip 9	1.84 ± 0.2^ab^	1.6 ± 0.178^ab^	1.38 ± 0.208^b^	2.3 ± 0.337^a^	0.677	0.155	0.021
Lip 20	1.06 ± 0.129	1.05 ± 0.229	1.1 ± 0.175	1.09 ± 0.0886	0.818	0.965	0.998
Lip13a+Lip13b	5.05 ± 0.323	4.67 ± 0.583	4.31 ± 0.441	9.36 ± 2.73	0.186	0.093	0.053

### Perturbation of fatty acid, TCA intermediate, and amino acid profiles in the brains of LDLr -/- and/or western diet fed mice

Brains from a subset of LDLr -/- and WT, mice fed either a CD or WD were subjected to metabolic analysis by GC/MS and LC/MS/MS to determine fatty acid, cholesterol, TCA cycle intermediate, and amino acid profiles (Table 4). Genotype did not alter C14:0 (myristic acid) content in the brains but exposure to a WD increased C14:0 (*p*≤0.05) and there was a trend for a diet x genotype interaction (*p* = 0.054). In addition, there was a diet effect with respect to C16:1 content which was elevated in WD compared to CD group. A dietary effect on cholesterol profiles was observed in the WT group but not in the LDLr -/- group, as the WD-fed WT mice had significantly higher value than the CD group.

**Table 4:** Shift in brain fatty acid and TCA cycle intermediate abundance with western diet and/or LDLr -/-. C57BL/6 (WT) or LDLr-/- mice were fed a control (CD) or western (WD) diet for 12 weeks and their brain fatty acid and TCA cycle intermediates were measured. Values are mean in μmol/g tissue ±SEM. Means in a row without a common subscript letter differ (p≤ 0.05) as analyzed by two-way ANOVA and Tukey test. GxD = Genotype x Diet interaction effect (n = 4/grp).

Fatty Acids	WT		LDLr -/-		*P*-value		
	CD	WD	CD	WD	Genotype	Diet	G×D^1^
C14	1.62 ± 0.207^ab^	1.63 ± 0.123^ab^	1.35 ± 0.0777^b^	1.97 ± 0.134^a^	0.825	0.048	0.054
C16	264 ± 21.6	288 ± 5.08	277 ± 13.2	272 ± 10	0.921	0.527	0.305
C16:1	0.884 ± 0.0756^b^	1.13 ± 0.0512^a^	0.969 ± 0.0373^ab^	1.06 ± 0.0586^ab^	0.925	0.013	0.192
C18	288 ± 18.7	327 ± 10.2	317 ± 6.19	321 ± 10.1	0.36	0.103	0.175
C18:1	211 ± 27.2	212 ± 13.9	187 ± 16.9	174 ± 13	0.123	0.752	0.724
C18:2	2.2 ± 0.379	2.27 ± 0.388	2.07 ± 0.23	1.78 ± 0.0902	0.321	0.71	0.566
C 20	2.83 ± 0.307	3.7 ± 0.292	3.15 ± 0.429	3.09 ± 0.203	0.667	0.226	0.17
C20:1	11 ± 2.24	10.9 ± 0.735	8.74 ± 0.725	8.76 ± 0.462	0.102	0.976	0.962
C22	0.902 ± 0.152	0.992 ± 0.0792	0.864 ± 0.127	0.881 ± 0.0508	0.511	0.635	0.744
Cholesterol	55.8 ± 8.73^b^	93.7 ± 7.76^a^	88.9 ± 7.31^a^	81.5 ± 7.55^ab^	0.208	0.076	0.014
**TCA Cycle intermediates**							
Succinate	0.518 ± 0.0323^a^	0.355 ± 0.0274^b^	0.351 ± 0.0192^b^	0.386 ± 0.0325^b^	0.034	0.043	0.004
BHB	0.024 ± 0.00339^b^	0.0358 ± 0.00452^ab^	0.0345 ± 0.00259^ab^	0.0414 ± 0.00249^a^	0.033	0.016	0.478
GABA	4.66 ± 0.708	5.59 ± 0.743	3.84 ± 0.611	6.43 ± 0.504	0.982	0.019	0.223
Fumerate	0.591 ± 0.058	0.535 ± 0.0272	0.512 ± 0.0349	0.492 ± 0.0199	0.134	0.336	0.645
Malate	0.131 ± 0.00695	0.13 ± 0.0018	0.123 ± 0.00439	0.137 ± 0.00635	0.946	0.252	0.163
Aspartate	2.05 ± 0.0786^bc^	2.7 ± 0.134^a^	1.81 ± 0.137^c^	2.49 ± 0.201^ab^	0.143	0.001	0.91
2-Hydroxyglutarate	0.213 ± 0.00918^b^	0.272 ± 0.0143^a^	0.202 ± 0.0142^b^	0.271 ± 0.00987^a^	0.622	<0.001	0.677
Glutamate	16.6 ± 1.81	19.2 ± 1.34	18.2 ± 1.99	21.7 ± 0.989	0.224	0.081	0.779
Glutamine	6.46 ± 0.287^bc^	7.63 ± 0.363^a^	5.78 ± 0.205^c^	7.29 ± 0.159^ab^	0.077	<0.001	0.531
Citrate	0.131 ± 0.0309	0.136 ± 0.0131	0.119 ± 0.0172	0.125 ± 0.0126	0.585	0.803	0.986
Lactate	12.9 ± 0.828^b^	17.8 ± 1.34^a^	16.1 ± 0.825^ab^	17.5 ± 0.963^a^	0.185	0.009	0.112
**Acyl-CoA’s**							
Malonyl CoA	1.07 ± 0.144	1.23 ± 0.134	1.12 ± 0.119	1.25 ± 0.0971	0.751	0.293	0.914
Acetyl CoA	3.25 ± 0.217^bc^	4.01 ± 0.235^ab^	4.23 ± 0.181^a^	3.02 ± 0.169^c^	0.991	0.294	<0.001
Succinyl CoA	2.57 ± 0.287	2.93 ± 0.548	2.5 ± 0.404	3.36 ± 0.354	0.669	0.158	0.556
Butyryl CoA	1.01 ± 0.0306^ab^	1.11 ± 0.0198^a^	1.09 ± 0.0158^a^	0.986 ± 0.0239^b^	0.327	0.85	0.001
Propionyl CoA	0.171 ± 0.00543	0.191 ± 0.00179	0.188 ± 0.00511	0.172 ± 0.00682	0.86	0.725	0.004
HMG CoA	1.01 ± 0.0335	1.13 ± 0.0257	1.07 ± 0.0434	1.01 ± 0.039	0.415	0.437	0.022
BHB CoA	0.0788 ± 0.00618	0.105 ± 0.00713	0.0962 ± 0.0108	0.0727 ± 0.00538	0.495	0.945	0.01
C16 0 CoA	0.851 ± 0.107	0.857 ± 0.136	0.876 ± 0.069	0.868 ± 0.149	0.883	0.994	0.95
C18 1 CoA	0.324 ± 0.0709	0.363 ± 0.0704	0.371 ± 0.0505	0.343 ± 0.0717	0.843	0.927	0.623
C20 4 CoA	0.242 ± 0.0258	0.266 ± 0.0237	0.383 ± 0.0449	0.262 ± 0.0765	0.176	0.328	0.155
AcAc CoA	0.000871 ± 0.000157	0.000713 ± 3.95e-05	0.00084 ± 6.9e-05	0.00103 ± 0.000342	0.533	0.88	0.415

Many of the TCA intermediates and amino acids measured were affected by diet. Those with the most significant changes include; aspartate, GABA, 2-hydroxyglutarate (reduced α-ketoglutarate) and glutamine, as these intermediates were increased with WD irrespective of genotype. Additionally, succinate decreased with WD intervention in the WT but was not altered by diet in the LDLr -/- mice. β-hydroxybutyrate (BHB) was significantly increased with WD irrespective of genotype, and the LDLr -/- were significantly elevated over the WT group demonstrating a genotype effect. Lactate was significantly elevated in the WD groups; whereas the LDLr -/- genotype appeared to have no effect, as they were similar irrespective of diet. Diet or genotype had no effect on fumarate, malate, glutamate, or citrate.

Acyl-CoA concentrations were significantly affected by WD and/or LDLr mutation. Acetyl-CoA, propionyl-CoA, butyryl-CoA, and BHB-CoA (intermediates of fatty acid oxidation) were significantly lower in the LDLr -/- group fed the WD compared to their diet matched WT groups. Although there were no significant differences in total-CoA concentrations with diet or genotype, the LDLr -/- group fed the WD trended lower compared to the WT WD group. C16:0-CoA, C18:0-CoA, C18:1-CoA, and C20:4 were not significantly different but C20:4 was significantly higher in the LDLr -/- group CD group. Although there were no significant changes in malonyl-CoA (intermediate of fatty acid synthesis and regulator of carnitine acyl-transport system for fatty acid transport into mitochondria) with diet or genotype, there was a decrease in the BHB-CoA to malonyl-CoA ratio in the LDLr -/- group fed the WD as compared to CD. This was likely due to the significant decrease in BHB-CoA.

Complex lipid analysis by CSH-ESI QTOF MS/MS demonstrated that PC moieties 32:0–3, 36:3B, 38:4A, and 38:5A are all elevated in LDLr-/- while PC 30:0, 35:1, 38:1 in addition to diacylglycerol, and glucosylceramides 40:1 and 42:1 were elevated with a WD (negatively charged CSH, Table 5). PC moiety’s 31:0 and 33:1 were the only ones affected by both WD and LDLr-/- and PC 31:0 showed a significant additive effect for diet and genotype. Further, ceremide 42:1(Cer18:1/24:0) and 42:2A (Cer18:1/24:1) in addition to fatty acid 18:1 and 20:1 were also found to be raised with a WD (Table 6, positively charged CSH). However, there were no significant changes in measured phosphatidylethanolamines, sphingomyelins or positively charged phosphatidylcholine moieties.

**Table 5:** Shift in negatively charged complex lipids with western diet and/or LDLr -/-. C57BL/6 (WT) or LDLr-/- mice were fed a control (CD) or western (WD) diet for 12 weeks and their brain complex lipids were measured by LC CSH-(+)ESI QTOF MS. Values are mean in μmol/g tissue ±SEM. Means in a row without a common subscript letter differ (p≤ 0.05) as analyzed by two-way ANOVA and Tukey test. GxD = Genotype x Diet interaction effect (n = 4/grp).

Complex Lipids (neg)	WT		LDLr -/-		*P*-value		
	CD	WD	CD	WD	Genotype	Diet	G×D^1^
Acylcarnitine C16:0	2040 ± 344	2410 ± 298	2490 ± 56.9	2660 ± 227	0.197	0.305	0.709
Acylcarnitine C18:1	1840 ± 422	2450 ± 225	2320 ± 263	2620 ± 162	0.28	0.135	0.604
Ceramide (d42:2)	749 ± 142	1350 ± 244	1100 ± 110	1150 ± 222	0.682	0.108	0.17
Cholesterol	56000 ± 1300	57200 ± 1410	55700 ± 1230	54800 ± 1000	0.285	0.885	0.422
DG (34:1)	693 ± 125	1440 ± 244	835 ± 274	1100 ± 74.7	0.628	0.024	0.246
GlcCer (d40:1)	18300 ± 1600	35900 ± 8130	19900 ± 1450	22100 ± 2480	0.194	0.043	0.105
GlcCer (d42:1)	36900 ± 1960	69300 ± 14600	38700 ± 3850	44400 ± 6610	0.189	0.041	0.134
GlcCer (d42:2)	133000 ± 10700	279000 ± 72100	139000 ± 10800	155000 ± 24400	0.154	0.06	0.12
LPC (16:0)	4930 ± 487	5540 ± 616	5020 ± 203	5540 ± 390	0.917	0.232	0.92
LPC (18:0)	2460 ± 161	2990 ± 338	2620 ± 220	2900 ± 50.2	0.853	0.088	0.582
LPC (18:1)	1450 ± 130	1840 ± 252	1510 ± 61.1	1690 ± 37.2	0.751	0.073	0.476
PC (30:0)	102000 ± 11700^b^	135000 ± 20100^ab^	118000 ± 8530^ab^	162000 ± 8580^a^	0.136	0.012	0.694
PC (31:0)	3810 ± 336^c^	5800 ± 519^b^	4550 ± 274^bc^	8800 ± 628^a^	0.002	<0.001	0.031
PC (32:0)	1280000 ± 122000^ab^	1060000 ± 76100^b^	1570000 ± 65900^a^	1540000 ± 96100^a^	0.001	0.205	0.325
PC (32:1)	188000 ± 25200	175000 ± 25000	245000 ± 22800	229000 ± 15900	0.029	0.528	0.939
PC (32:3)	3730 ± 320^b^	4050 ± 317^ab^	4170 ± 221^ab^	5310 ± 528^a^	0.038	0.067	0.28
PC (33:1)	4400 ± 357^b^	6490 ± 666^ab^	5630 ± 277^b^	8670 ± 709^a^	0.008	<0.001	0.387
PC (34:0)	224000 ± 13900	243000 ± 38000	256000 ± 22400	281000 ± 14200	0.173	0.375	0.916
PC (34:1)	1820000 ± 23900	1800000 ± 36900	1850000 ± 11600	1860000 ± 7080	0.073	0.81	0.543
PC (35:1)	10900 ± 660	17400 ± 3480	11200 ± 835	17400 ± 612	0.928	0.005	0.937
PC (36:1)	483000 ± 47500	631000 ± 121000	502000 ± 33400	583000 ± 30200	0.838	0.123	0.632
PC (36:2)	171000 ± 10900	194000 ± 31300	181000 ± 10800	198000 ± 3510	0.691	0.284	0.882
PC (36:3) B	9830 ± 1380^ab^	7210 ± 1700^b^	13000 ± 1150^ab^	13800 ± 1330^a^	0.004	0.529	0.247
PC (36:4) B	232000 ± 38900	177000 ± 21600	277000 ± 29500	280000 ± 45400	0.056	0.472	0.426
PC (36:5) B	1280 ± 136	1000 ± 37.6	1420 ± 251	1300 ± 74.1	0.153	0.24	0.627
PC (38:1)	13500 ± 1330	28100 ± 7130	12300 ± 1110	14900 ± 1790	0.081	0.041	0.137
PC (38:2)	15000 ± 875	26000 ± 6360	15100 ± 1060	17900 ± 1680	0.26	0.062	0.245
PC (38:3)	6000 ± 448	7950 ± 1860	6800 ± 788	8150 ± 387	0.645	0.141	0.782
PC (38:4) A	309000 ± 39200	252000 ± 26400	343000 ± 27600	357000 ± 28500	0.044	0.498	0.274
PC (38:5) A	46800 ± 7670	37800 ± 3160	57600 ± 6080	57500 ± 6870	0.03	0.48	0.483
PC (38:5) B	4580 ± 1250	4380 ± 426	5890 ± 358	5810 ± 1750	0.24	0.902	0.958
PC (38:6) A	635000 ± 38400	682000 ± 65200	682000 ± 36600	691000 ± 55200	0.589	0.586	0.709
PC (38:6) C	961 ± 20.5	931 ± 72.4	955 ± 174	1100 ± 45.9	0.408	0.552	0.377
PC (39:6)	1040 ± 220	1660 ± 123	1060 ± 264	1510 ± 97.9	0.722	0.015	0.64
PC (40:4)	9480 ± 918	11500 ± 2750	10100 ± 1020	12400 ± 1030	0.658	0.212	0.924
PC (40:5) A	11700 ± 809	11400 ± 2710	12600 ± 809	13400 ± 492	0.35	0.865	0.703
PC (40:5) B	3100 ± 227	2820 ± 789	2720 ± 280	3910 ± 305	0.457	0.337	0.137
PC (40:6) A	1390 ± 165	1450 ± 145	1600 ± 134	1580 ± 162	0.271	0.897	0.807
PC (40:6) B	189000 ± 24100	218000 ± 41000	170000 ± 23900	194000 ± 29900	0.497	0.396	0.928
PC (40:7)	266000 ± 12700	331000 ± 46300	297000 ± 26300	312000 ± 29800	0.857	0.225	0.428
PC (40:8)	891 ± 222	1120 ± 68.6	1140 ± 282	1090 ± 325	0.663	0.735	0.566
PC (o-32:0)	6010 ± 981^ab^	4790 ± 627^b^	7120 ± 468^ab^	8260 ± 978^a^	0.014	0.961	0.162
PC (o-34:0)	2020 ± 111	1910 ± 179	2140 ± 114	2340 ± 205	0.106	0.746	0.339
PE (34:1)	28400 ± 2030	33600 ± 4730	29100 ± 1260	31600 ± 1240	0.811	0.178	0.632
PE (36:1)	42600 ± 3870	56700 ± 9250	42200 ± 1410	47100 ± 3660	0.369	0.105	0.411
PE (36:2)	40900 ± 2570	54300 ± 8810	40100 ± 2390	43800 ± 3600	0.288	0.119	0.36
PE (38:4)	179000 ± 25800	152000 ± 16800	191000 ± 14300	206000 ± 14200	0.096	0.739	0.278
PE (38:5)	33100 ± 1200	38800 ± 4500	32600 ± 1510	35400 ± 1370	0.462	0.121	0.574
PE (38:6)	222000 ± 20700	224000 ± 24300	275000 ± 8730	269000 ± 14800	0.019	0.932	0.835
PE (40:4)	23800 ± 3770	21900 ± 3370	25900 ± 1240	28600 ± 2590	0.154	0.886	0.448

**Table 6:** Shift in positively charged complex lipids with western diet and/or LDLr -/-. C57BL/6 (WT) or LDLr-/- mice were fed a control (CD) or western (WD) diet for 12 weeks and their complex lipids were measured by LC CSH- (-) ESI QTOF MS. Values are mean in μmol/g tissue ±SEM. Means in a row without a common subscript letter differ (p≤ 0.05) as analyzed by two-way ANOVA and Tukey test. GxD = Genotype x Diet interaction effect (n = 4/grp).

Complex Lipids (pos)	WT		LDLr-/-		*P*-value		
	CD	WD	CD	WD	genotype	diet	G×D^1^
Ceremide 34:1	14700 ± 1730	19300 ± 2980	15200 ± 2020	19000 ± 3340	0.967	0.136	0.885
Ceremide 34:2	1500 ± 398	677 ± 204	1330 ± 477	1740 ± 343	0.255	0.58	0.12
Ceremide 36:1	505000 ± 64300	535000 ± 60400	516000 ± 41700	568000 ± 55900	0.704	0.479	0.852
Ceremide 38:1	47600 ± 1080	46300 ± 3680	45400 ± 2830	46800 ± 2730	0.766	0.997	0.629
Ceremide 39:1	5210 ± 886	5090 ± 575	5530 ± 364	5910 ± 851	0.435	0.862	0.728
Ceremide 40:1	10500 ± 930	14000 ± 2110	10300 ± 774	12600 ± 1710	0.571	0.076	0.699
Ceremide 40:2	1340 ± 337	2340 ± 404	1810 ± 598	2320 ± 401	0.631	0.117	0.599
Ceremide 41:1	5740 ± 677	7530 ± 1260	5880 ± 381	6810 ± 686	0.725	0.12	0.605
Ceremide 42:1	5950 ± 623	9170 ± 1660	6130 ± 532	8140 ± 1540	0.732	0.05	0.626
Ceremide 42:2A	28000 ± 2870	46800 ± 8970	29100 ± 4290	36500 ± 5880	0.458	0.048	0.356
Ceremide 42:2B	986 ± 147	1790 ± 322	1350 ± 27.2	1810 ± 294	0.506	0.122	0.616
FA 18:1	165000 ± 27000	261000 ± 45800	178000 ± 20800	222000 ± 29400	0.684	0.049	0.429
FA 20:1	20400 ± 2580^ab^	37800 ± 7430^a^	18300 ± 2110^b^	23000 ± 3020^ab^	0.076	0.025	0.169
FA 20:2	1380 ± 209	1880 ± 385	1140 ± 134	1700 ± 244	0.435	0.064	0.889
FA 20:3	5620 ± 1210	7210 ± 1060	5410 ± 915	7920 ± 996	0.816	0.074	0.668
FA 20:3	5670 ± 880	8050 ± 1210	6410 ± 765	6990 ± 830	0.87	0.139	0.358
FA 20:4	464000 ± 51300	497000 ± 50600	524000 ± 45600	563000 ± 36100	0.2	0.45	0.942
FA 20:5	1070 ± 380	1470 ± 414	1920 ± 314	2060 ± 349	0.072	0.476	0.727
FA 22:0	4350 ± 1560	3280 ± 1630	6110 ± 937	5490 ± 1690	0.205	0.581	0.88
FA 22:6	85000 ± 10200	127000 ± 18200	95400 ± 8080	110000 ± 18900	0.844	0.076	0.378
FA 24:1	3760 ± 687^ab^	6760 ± 1530^a^	2910 ± 375^b^	3660 ± 572^ab^	0.05	0.061	0.239
GlcCer 38:1	22800 ± 2140	35400 ± 6160	23600 ± 1940	25600 ± 3160	0.251	0.073	0.182
GlcCer 40:1	107000 ± 9480	162000 ± 27200	110000 ± 6680	118000 ± 13100	0.227	0.076	0.176
GlcCer 41:1	106000 ± 7200	170000 ± 31500	104000 ± 7380	116000 ± 14500	0.146	0.055	0.18
GlcCer 42:1	307000 ± 18400	520000 ± 102000	306000 ± 28500	338000 ± 52100	0.15	0.061	0.153
GlcCer 42:2	840000 ± 56000	1180000 ± 189000	813000 ± 46700	862000 ± 116000	0.166	0.122	0.237
GlcCer d14:1/20:0(2OH	7510 ± 2470^ab^	3540 ± 1850^b^	7640 ± 2100^ab^	13900 ± 777^a^	0.018	0.565	0.02
LPC 16:0	10800 ± 1310	12000 ± 1520	10900 ± 508	11800 ± 870	0.938	0.358	0.908
LPC 18:0	6700 ± 585	7710 ± 903	6460 ± 216	7170 ± 492	0.529	0.175	0.805
LPC 18:1	2800 ± 203	3610 ± 472	2930 ± 82.2	3280 ± 377	0.765	0.096	0.5
LPE 16:0	1880 ± 131	2260 ± 249	2070 ± 123	2700 ± 526	0.326	0.125	0.686
LPE 18:0	6560 ± 718	7830 ± 996	7110 ± 487	9630 ± 2160	0.371	0.16	0.63
LPE 22:6	12500 ± 3620	8770 ± 552	9090 ± 470	7230 ± 1200	0.229	0.179	0.644
PC 32:0	271000 ± 25300^ab^	224000 ± 14200^b^	318000 ± 14900^a^	304000 ± 15800^a^	0.004	0.12	0.375
PC 32:1	18700 ± 3280	17600 ± 2670	21300 ± 1470	20600 ± 624	0.246	0.695	0.93
PC 33:1	1960 ± 851	4870 ± 309	3190 ± 1000	4110 ± 1110	0.795	0.049	0.276
PC 34 0	80200 ± 3220	75300 ± 5860	84400 ± 5240	85700 ± 2420	0.124	0.697	0.498
PC 34:1	342000 ± 28600	344000 ± 29300	356000 ± 15200	364000 ± 6760	0.454	0.822	0.895
PC 34:2	8480 ± 317	6830 ± 1630	9780 ± 500	9740 ± 490	0.038	0.367	0.39
PC 36:1	156000 ± 10800	178000 ± 19400	151000 ± 7220	160000 ± 9320	0.385	0.234	0.588
PC 36:2	28200 ± 1930	31900 ± 3310	28900 ± 1200	31000 ± 840	0.941	0.181	0.696
PC 36:3 B	4260 ± 463	3880 ± 233	4890 ± 315	3850 ± 1230	0.674	0.321	0.637
PC 36:4 B	67700 ± 8200	50400 ± 3050	71000 ± 3250	69900 ± 5910	0.062	0.119	0.169
PC 38:2	6350 ± 134	9180 ± 1630	5940 ± 384	6330 ± 747	0.102	0.106	0.209
PC 38:3	2260 ± 60.3	2420 ± 194	2230 ± 106	2240 ± 66.4	0.399	0.476	0.532
PC 38:4 A	45400 ± 5790	36500 ± 2050	47300 ± 2990	48000 ± 2230	0.088	0.276	0.203
PC 38:5 A	17700 ± 2350	14200 ± 784	19200 ± 1170	18600 ± 1240	0.074	0.2	0.359
PC 40:6 B	32500 ± 4090	40700 ± 3790	27800 ± 2610	30200 ± 3430	0.051	0.158	0.417
PC 40:7	42200 ± 4110	52300 ± 7920	45000 ± 2490	48900 ± 4150	0.954	0.195	0.551
PE 34:1	75300 ± 7710	80300 ± 9310	73200 ± 4210	77500 ± 3510	0.716	0.5	0.958
PE 36:2	92800 ± 5240	120000 ± 17200	86400 ± 4960	94700 ± 10200	0.165	0.123	0.399
PE 36:4	46700 ± 6390	39300 ± 1850	48900 ± 3720	48500 ± 2120	0.175	0.339	0.387
PE 38 2	838 ± 77.7	1130 ± 270	958 ± 96.7	1180 ± 115	0.697	0.147	0.839
PE 38:4 A	24600 ± 5950	25000 ± 2410	29600 ± 2020	24500 ± 6290	0.638	0.616	0.559
PE 38:4 B	444000 ± 64100	363000 ± 29200	460000 ± 32400	482000 ± 24100	0.121	0.475	0.227
PE 38:5	84400 ± 5320	94200 ± 7980	81900 ± 3580	88700 ± 3570	0.473	0.152	0.792
PE 38:6	819000 ± 124000	728000 ± 94300	964000 ± 48500	921000 ± 30300	0.064	0.437	0.777
PE 40:4	158000 ± 28100	139000 ± 24500	157000 ± 12200	171000 ± 18500	0.498	0.904	0.465
PE 40:6	1200000 ± 70300	1110000 ± 54200	1270000 ± 46900	1250000 ± 31100	0.064	0.32	0.482
PE 44:10	413000 ± 52500	468000 ± 74700	392000 ± 35200	459000 ± 36500	0.773	0.265	0.908
PG 32:0	12200 ± 1850	11800 ± 1430	10300 ± 552	11400 ± 559	0.357	0.81	0.549
PG 34:1	54800 ± 1980	57700 ± 5120	49800 ± 5130	54100 ± 4900	0.355	0.437	0.89
PI 36:4	3480 ± 1550	3200 ± 1400	6010 ± 68.3	6030 ± 222	0.026	0.904	0.886
PI 38:5	3620 ± 264	2960 ± 214	3510 ± 288	3670 ± 173	0.236	0.327	0.114
PS 36:2	43700 ± 1640	51100 ± 5040	39600 ± 2610	44300 ± 3130	0.128	0.096	0.691
SM d34:1	9590 ± 194	11200 ± 1290	8620 ± 477	10200 ± 555	0.212	0.056	0.992
SM d36:1	80000 ± 3370	71000 ± 7090	67000 ± 4770	75500 ± 3940	0.413	0.966	0.106
SM d36:2	16900 ± 3770	12200 ± 2600	15500 ± 303	18700 ± 2320	0.34	0.764	0.147
SM d38:1	8560 ± 291	10500 ± 872	7750 ± 614	8480 ± 807	0.059	0.073	0.387
SM d40:1	24500 ± 7000	31100 ± 9540	25600 ± 6590	17000 ± 7480	0.415	0.898	0.345
SM d41:1	11500 ± 3030	20200 ± 3580	13400 ± 780	13600 ± 3910	0.459	0.175	0.186
SM d42:2A	40300 ± 2120	58400 ± 10800	37100 ± 2320	43300 ± 6240	0.18	0.083	0.374
SM d42:3	856 ± 221	1440 ± 372	868 ± 254	1150 ± 260	0.746	0.164	0.613
SM d44:2	1420 ± 123	1510 ± 352	1130 ± 262	1410 ± 206	0.436	0.479	0.708

### Oxylipins and endocannabinoids abundance segregates mice groups by diet or genotypes with a shift to a pro-inflammatory state

**Fig 6:**
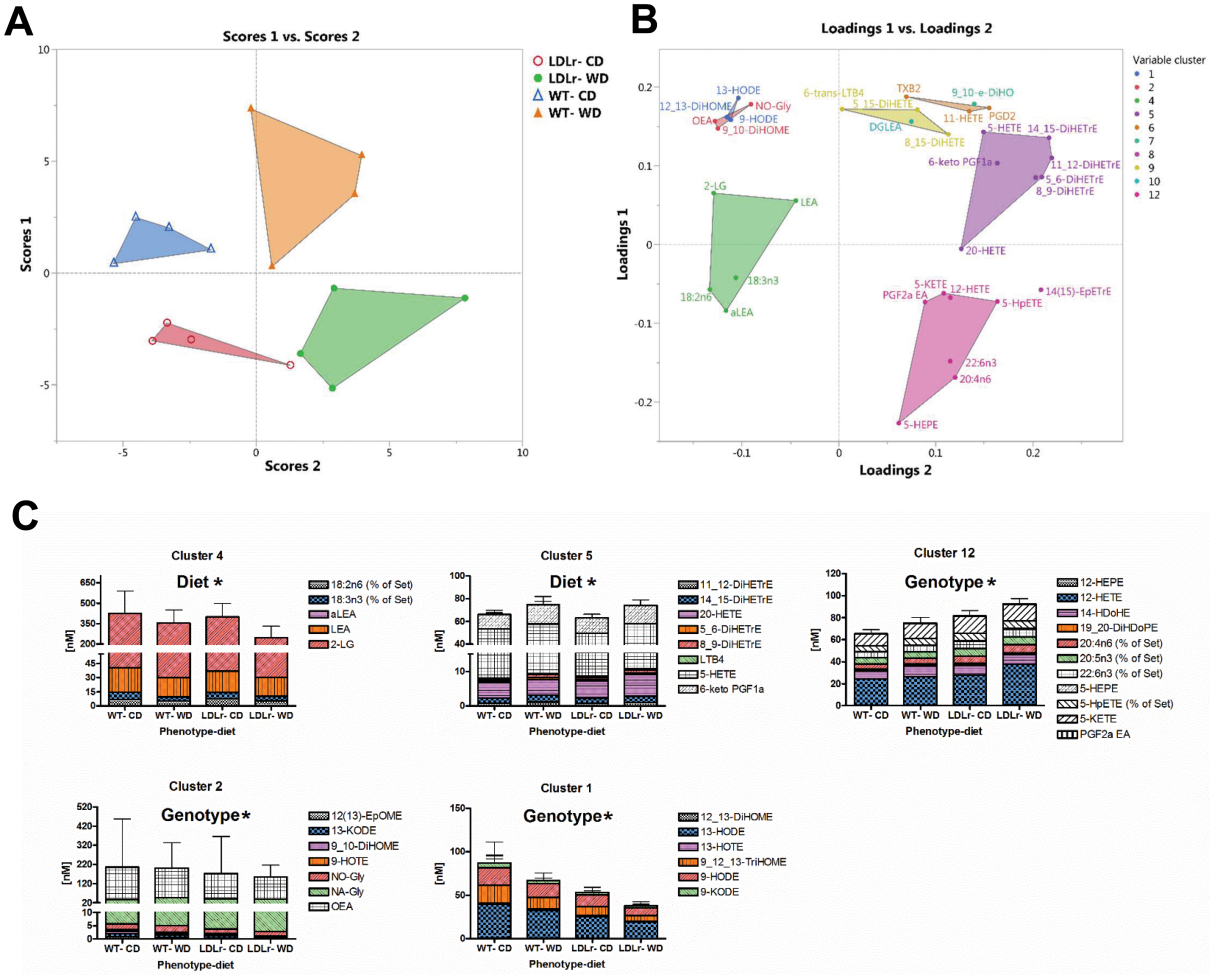
Shift in brain free oxylipins, endocannabinoids and polyunsaturated fatty acids (PUFA) to a proinflammatory profile by diet. Partial least squares analysis (A) discriminate between C57BL/6 (WT) or LDLr-/ mice, fed a control (CD) or western (WD) diet due to differences in brain metabolite pattern seen in associated loading plot (B). Hierarchical cluster analysis categorized measured variables into clusters and ANOVA analysis tested for effect of the diet, genotype, and the diet x genotype interaction. Clusters identified as having either a significant change are shown (C). Loading plot: variables with VIP scores >1 are shown. The variables are colored according to their clusters affiliations.

Partial least square analysis, together with the mixed model ANOVA, were applied to determine the impact of experimental groups on the level of free oxylipins, endocannabinoids and polyunsaturated fatty acids (PUFAS) (Fig 6A–6C). Forty seven percent of variables had VIP score greater than 1, suggesting that observed differences are most likely not false positives. Major diet and genotype effect were observed, however, without significant interaction between them. WD groups were characterized by decreased levels of 18 carbon PUFAs (alpha linolenic acid (aLA) and linoleic acid (LA)) as well as their corresponding ethanol amines (aLEA and LEA). Additionally, WD elevated the levels of soluble epoxide hydrolase metabolites of arachidonic acid (DiHETrEs) as well as 5- lipoxygenase (LOX) metabolites (LTB4 and 5- Hydroxyicosatetraenoic acid (HETE)) and COX metabolites (20-HETE and 6-keton PGF1a). LDLr -/- animals showed increased level of long chain (C 20 and 22) PUFAs as well as their 12-LOX metabolites (12-HETE, 12 HEPE, 14-HDoHE). Moreover, LDLr -/- decreased the levels of 18 carbon derived oxylipins (from both LOX and CYP pathways). Interestingly, the levels of their parent PUFAs were not impacted by the LDLr -/-.

### Western diet exacerbates LDLr-/- elevation of plasma lipid levels without significantly changing body weight

At the initiation of the diet, WT mice were heavier (23.5 ± 0.2g) than LDLr -/- (22.7 ± 0.2g; *p*≤ 0.05) mice. Mice of both genotypes (WT and LDLr -/-) fed a WD had a significant increase in body weight compared to CD fed, and the C57BL/6 WD-fed were significantly heavier than western fed LDLr -/- (Table 7, *p*< 0.001). A subset of mice (n = 4/grp) had their blood collected at termination and non-fasting glucose, insulin, and lipid content was measured. There was no change in glucose but a significant difference between insulin levels was found in CD and WD fed mice (668.6 ± 148.2 and 3187.0 ± 2263.2 pg/mL, *p*≤ 0.01). Total cholesterol, triglyceride levels, HDL and LDL levels were all found to have significantly effect with diet or genotype as well as having a diet x genotype interaction.

**Table 7:** Non-fasting physiological parameters at 20 weeks. Data are represented as mean ± SEM for wild type (WT) LDLr -/- on either a control (CD) or western (WD) diet, n = 20 for body weight and 4 for all other parameters. ^a-c^ Means in a row without a common superscript letter differ (P < 0.05) as analyzed by two-way ANOVA and the TUKEY test. G × D^1^ = Genotype × Diet interaction effect. n = 20 for body weight and 4 for all other parameters.

Physiological Parameter	WT		LDLr -/-		*P*-value		
	CD	WD	CD	WD	Genotype	Diet	G×D^1^
Body mass(g)	30.9 ± 0.45^b^	39.2 ± 0.809^a^	30.8 ± 0.538^b^	33 ± 0.605^b^	<0.001	<0.001	<0.001
Glucose (pg/dl)	286 ± 34	300 ± 15	303 ± 10.2	310 ± 15	0.533	0.617	0.879
Insulin (pg/ml)	620 ± 77.3	5320 ± 2420	718 ± 176	1060 ± 291	0.115	0.062	0.1
TG (mg/dl)	164 ± 14.1^b^	56.7 ± 11.7^b^	393 ± 69.6^b^	1300 ± 161^a^	<0.001	0.001	<0.001
TC (mg/dl)	153 ± 8.25^c^	273 ± 7.72^c^	567 ± 28.2^b^	2090 ± 75.8^a^	<0.001	<0.001	<0.001
HDL (mg/dl)	122 ± 8.31^b^	206 ± 7^b^	211 ± 8.91^b^	476 ± 45.8^a^	<0.001	<0.001	0.003
LDL (mg/dl)	31.6 ± 2.76^c^	64.3 ± 3.17^c^	489 ± 86.7^b^	1300 ± 46.4^a^	<0.001	<0.001	<0.001

### LDLr -/- and western diet increase aortic sinus plaque formation and hepatic lipidosis

**Fig 7:**
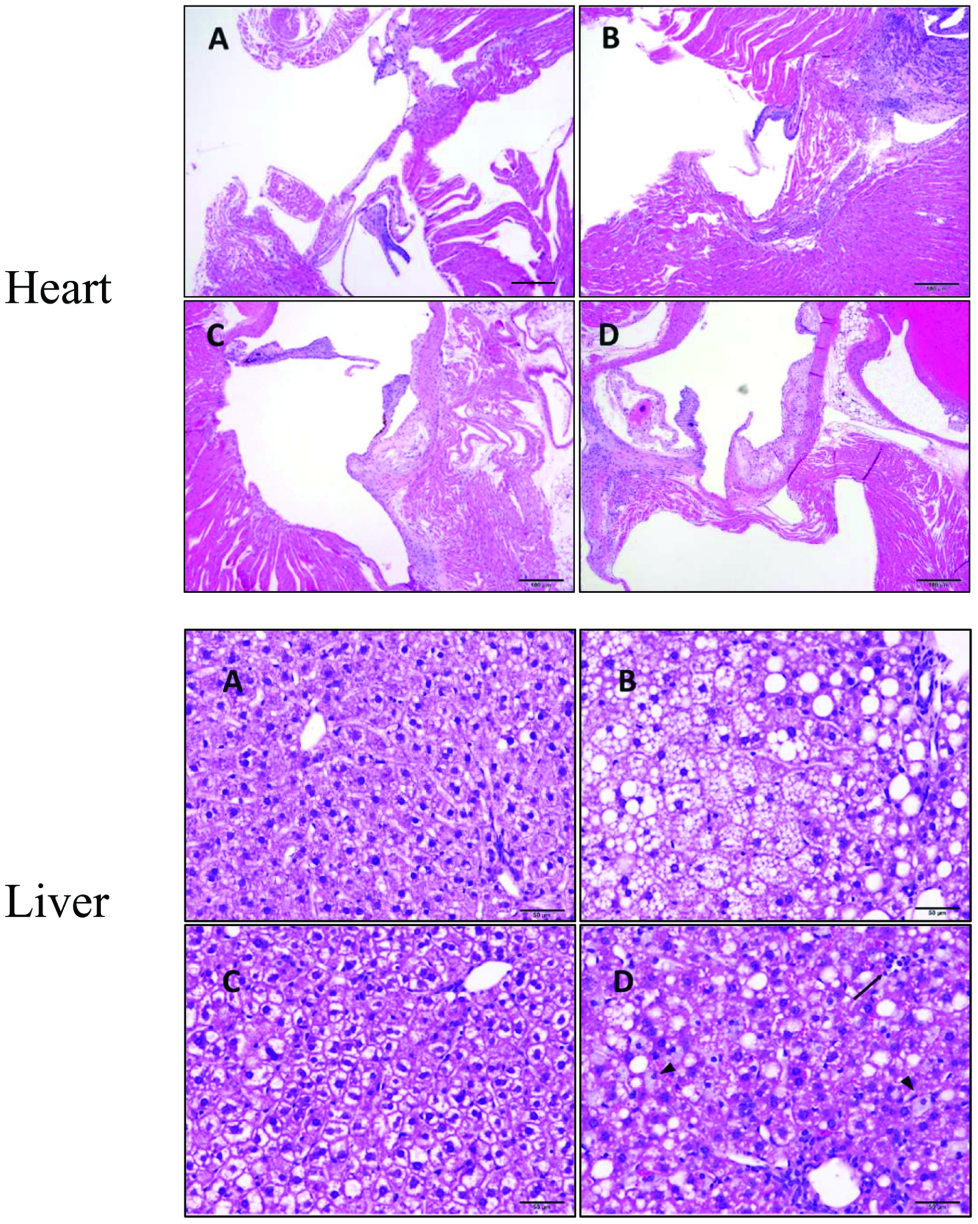
Cardiac and liver histopathology. In **heart**, CD (A) and WD-fed (B) wild type mice had normal aortic sinus structures, but the aortic sinus of LDLr -/- mice on CD had subendothelial foam cell plaques and medial accumulations of extracellular cholesterol (C), both were more extensive in LDLr -/- mice fed a WD (D). In **liver**, compared with CD fed (A), WT mice given a WD (B) had marked microvesicular and macrovesicular hepatic lipidosis that was most marked in the periacinar regions. CD fed LDLr -/-mice (C) had more hepatocellular glycogen than WT mice but were otherwise histologically normal. LDLr -/- mice given a WD (D) had generalized macrovesicular lipidosis, enlarged Kupffer cells with foamy cytoplasm (Arrowheads), periportal mononuclear inflammation, and individual hepatocyte necrosis accompanied by mixed inflammatory cell infiltrates (Arrow). Scale bar: heart = 100μM and liver = 50μM.

To determine the effects of diet and genotype on the general pathology of systemic organs, brain, liver, heart, kidney, pancreas, skeletal muscle, and lung were evaluated by routine histopathology. No gross changes in morphology and no significant lesions were seen in brain, kidney, pancreas, skeletal muscle, and lung. However, diet and genotype related lesions were present in heart and liver. LDLr -/- mice fed CD had foam cell plaques on the endocardial surface of the aortic sinus (Fig 7, heart). These focal accumulations were composed principally of aggregates of large mononuclear cells with cytoplasm filled with vacuoles. The aortic sinus of LDLr -/- mice fed WD also contained foam cell plaques but these were more extensive and foam cells were interspersed with extracellular accumulations of acicular clefts characteristic of cholesterol deposits, increased extracellular matrix, and scattered small mononuclear inflammatory cells (complex atheroma). Both WT and LDLr -/- mice fed a WD had marked hepatic lipidosis (Fig 7, liver). In WT mice, a zonal accumulation of lipid in hepatocytes progressed from multiple lipid droplets in periacinar regions to larger single lipid accumulations in periportal regions. LDLr -/- mice fed WD had more prominent generalized hepatocyte lipid accumulations with randomly distributed individual necrotic hepatocytes and associated mononuclear inflammatory cell infiltration. In addition, Kuppfer cells were markedly enlarged due to microcystic intracytoplasmic vacuoles interpreted to represent lipid accumulation.

## Discussion

**Fig 8:**
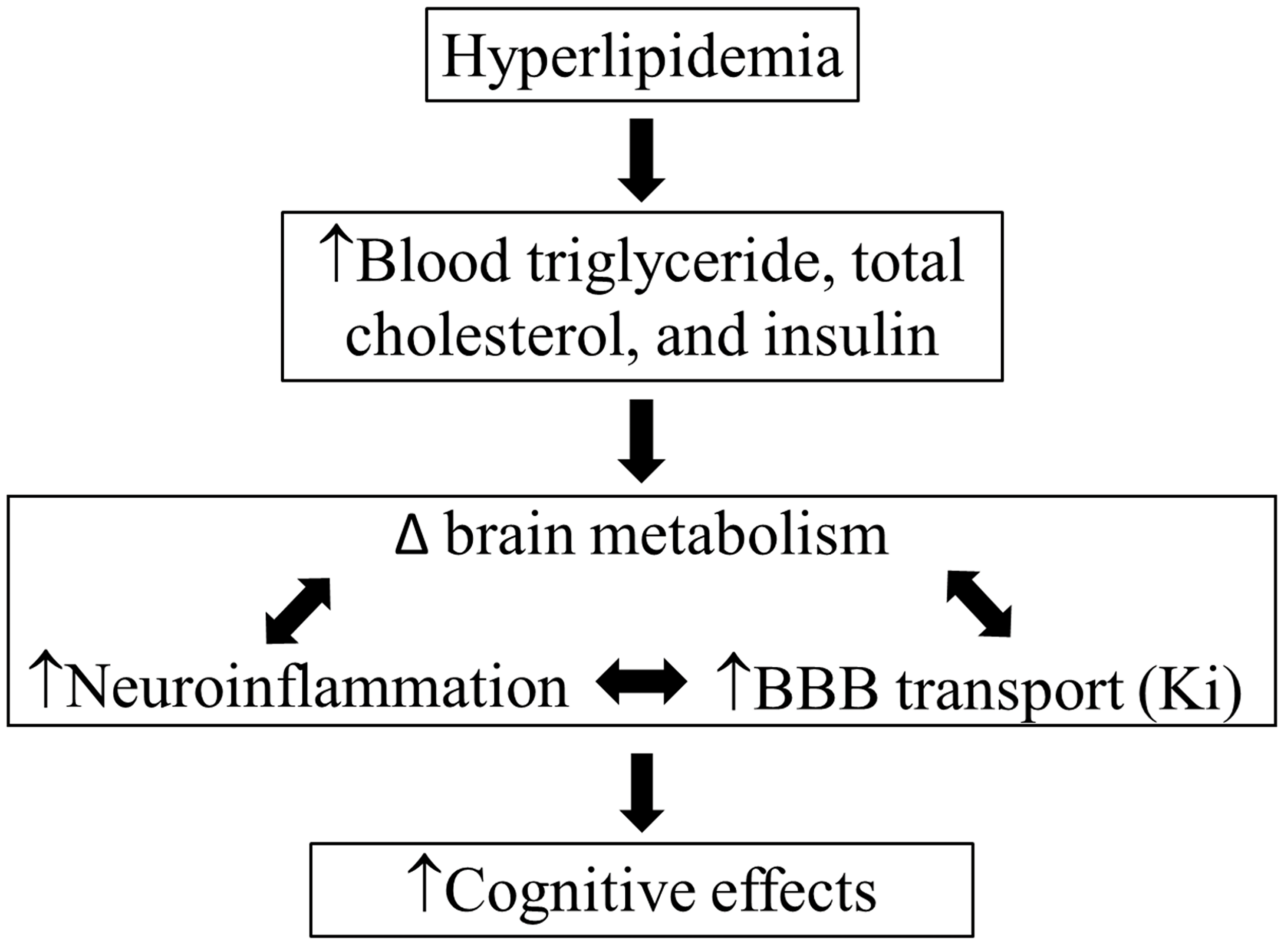
Model of physiological pathway in which diet or genotype induced hyperlipidemia alters cognitive function.

While there is significant epidemiologic evidence that a diet high in saturated fat and simple carbohydrates resulting in elevated plasma lipids and insulin resistance puts individuals at a greater risk for dementia and cognitive impairment, the cellular metabolic reasons for this are not fully understood. This study was conceived to better understand the mechanisms through which a western diet (WD—moderately high in saturated fat, sucrose, and cholesterol) induced cognitive impairment by assessing the brain molecular, cellular, biochemical, and physiological changes that occur. Our studies demonstrate that WD alters brain metabolism, activates microvessels and microglia, and increases BBB transport, all of which may be linked to the observed moderate altered cognitive function (Fig 8 and Tables 8 and 9).

**Table 8:** Functional changes by western diet (WD) or in a genetic (G) model of hyperlipidemia.

Parameter	Overall	Summary
**Cognitive function**		
Y-maze	↓ WD	Decrease % spontaneous alterations in WD
MWM	↑ WD	Increase % time in platform quadrant in WD in probe trial
RAWM	↓ G	Increase in the number of Trial 4 errors in LDLr -/-
**BBB transport**		
Ki	↑ WD	Increased in WD
**IHC**		
Factor VIII	↑ G x WD	Elevated in LDLr -/- WD (hippocampus & thalamus)
IBA1	↑ G x WD	Elevated in WT WD (cortex & hippocampus)
**Gene Expression (whole brain RT-PCR)**		
ATF3, IL-6, CD32 & CD206	↓G	Reduced in LDLr -/-
CD86	↓ WD	Reduced with WD
**Brain Metabolites (**^**1**^**H-MRS Spec)**		
glutamine	↓ G x WD	Reduced in LDLr -/- WD
lip13a+13b	↑ G x WD	Elevated in LDLr -/- WD
**Brain glucose utilization**		
18FDG-PET	↑ G	Increased Standardized Uptake Value in LDLr -/-
**Brain Metabolites (GC/MS & LC/MS)**		
C14	↑ WD	Elevated in WD
C16:1	↑ WD	Elevated in WD
Cholesterol	↑ G x WD	Elevated in WT WD and LDLr -/- WD
TCA cycle intermediates	↑ WD	Elevated BHB, GABA, Aspartate, 2-hydroxyguterate, Glutamine, Lactate in WD
Acyl-CoA's	↑↓ G x WD	Acetyl-CoA elevated in LDLr -/- CD, Acetyl- and Butyryl-CoA reduced in LDLr -/- WD
free 18 C PUFA’s	↓ WD	Reduced in WD
HETEs & leukotriene	↑ WD	Elevated in WD
**Physiological**		
Weight	↑ WD, G, and G x WD	Increased with WD, decrease with LDLr -/-
Insulin	↑ WD	Increased with WD
TG	↑ WD, G, and G x WD	Increased in LDLr -/- and WD fed LDLr -/-, but decreased in WD fed WT
TC	↑ WD, G, and G x WD	Increased with WD or LDLr -/-, highest in LDLr -/- WD
HDL	↑ WD & G	Increased with WD or LDLr -/-, highest in LDLr -/- WD
LDL	↑ G and G x WD	Increased with WD or LDLr -/-, highest in LDLr -/- WD

**Table 9:** Cardiac and liver histopathology with western diet and/or LDLr -/-.

Histology	WT CD	WT WD	LDLr -/- CD	LDLr-/- WD	Summary
Aortic sinus			+	++	foam cell & plaque formation
Liver		lipidosis	glycogen	lipidosis	
				enlarged, foamy Kupffer cells	
				inflammation/necrosis	

Previous studies have demonstrated that either a WD [29] and/or gene knockout-induced hyperlipidemia [9, 58] can result in cognitive impairment in mice while others have demonstrated a link of cognitive impairment to neuroinflammation or metabolic shifts [59, 60]. We extended these findings by comprehensively evaluating the consequences of WD and LDLr -/- genotype on behavior as an indicator of cognitive impairment. Both the MWM and the Y-maze alternation tests for cognition were sensitive to the effects of the WD with group sizes as small as 8 mice/group. While % of alternation triplets was decreased in WD-fed mice (an indicator of reduced cognitive function), these same animals showed an increase in % time in the platform quadrant on the probe trial for the MWM (indicating improved function). Both tests assess short-term spatial memory, but the MWM additionally evaluates day-to-day learning, which may contribute to the differences seen here. The Y-maze and MWM results were sensitive to WD, but they did not reflect an effect of LDLr-/- genotype on cognitive endpoints as seen by other groups [9]. Therefore, a second study was conducted where a different spatial learning and memory problem was presented on each of nine successive days of testing and number of errors on the last trial of each problem was compared across days. The RAWM detected an impairment in ability of LDLr -/- mice to solve a complex spatial problem with repeated exposure to different problems. Our studies thus indicate that Y-maze alternation can provide a rapid, inexpensive assessment of the cognitive effects of a WD. However, to avoid false negative findings in screening for cognitive deficits seen with LDLr-/- mutation more complex cognitive testing of the RAWM, are most sensitive and appropriate. These modest and potentially contradictory findings are not unexpected, as even genetic mouse models of Alzheimer’s (Tg2576) show cognitive impairments by T maze (modification of Y-maze) most consistently, followed by MWM and RAWM, but there is great variability between studies [61].

Even though under normal conditions the blood-brain barrier (BBB) is highly regulated, metabolic stresses such as hyperlipidemia can cause neurovascular unit dysfunction and increase BBB permeability [62]. Studies by our lab have demonstrated that a bolus infusion of TGRL lipolysis products, similar to an increase that would be found in the postprandial state, can lead to a transient increase in the BBB Gd-DTPA transfer coefficient (Ki) [17, 63]. Here Ki was increased in animals fed a WD, while cerebral blood flow, determined by perfusion weighted imaging, was essentially normal. Interestingly, despite higher circulating lipids on a CD, LDLr -/- mice did not have elevated baseline Ki and no additive effect of a WD beyond that of WT. Our findings are consistent with a hypothesis that chronic consumption of a WD increases microvascular leak, assuming no concurrent increase in capillary surface area, increases BBB permeability. Perhaps this is related to neuroinflammation, activation of endothelial apoptotic pathways [15, 64], or increased transcytosis. Regardless, an increase in BBB leak would likely increase movement of blood solutes including lipids and lipoproteins into the brain interstitial space which may alter neurovascular metabolism and inflammation.

High-fat diet induced obesity in C57BL/6 mice not only impaired hippocampus-dependent memory and reduced long-term potentiation (as determined by Y-maze and novel object recognition) but has also shown increased microglia activation and loss of synapses [19, 65, 66]. Our immunohistochemical analysis demonstrates increased relative surface area of IBA1 in western diet fed WT mice where most immunopositive cells had multiple prominent radiating elongate cytoplasmic processes typical of microglia. Since upregulation of IBA1is a marker of microglial activation [67–69], our results suggest neuroinflammation is increased by WD in WT but not LDLr -/- mice. However, it should be noted that our approach does not discriminate between increased numbers of microglial cells vs. upregulation of IBA1 in greater proportions of microglial processes.

Upregulation of factor VIII immunostaining in WD-fed LDLr -/- mice was an unexpected finding as our initial hypothesis was that a WD would decrease the density of microvessels in brain as is seen in individuals with Alzheimer’s disease [70, 71]. However, as with IBA1 staining, our approach does not distinguish between increased densities in pre-existing endothelial cells versus increased endothelial cell numbers. Interestingly other studies associated elevated circulating plasma factor VIII as a risk factor for vascular dementia [72] and was found to be significantly elevated in plasma of patients with other brain injury models including ischemic stroke [73]. Further liver disease is associated with elevated plasma factor VIII levels which is thought to be due to production by sinusoidal endothelial cells [74]. It is possible that the increase in brain microvascular factor VIII may be due to an increase in endocytosis by BBB endothelial cells. Whether microvascular endothelial cell activation as indicated by an increased expression of factor VIII relate to hypoxia related angiogenesis or endocytosis of circulating factor VIII remains to be determined as does any correlation of these variables with changes in BBB transfer coefficient.

CD16, CD32 & CD86 versus CD 206 & arginase expression have previously been used as markers of differentiation between M1 vs M2 microglia and microglia activation [56, 57]. The downregulation of CD86 with an increase in PTGS2 and IBA1 expression in WD fed mice may indicate a shift from M1- to M2- like phenotype as described by Abutbul et al. [75]. However, LDLr -/- showed a decrease in CD32 and CD206 (M1 and M2, respectively) and a decrease in IL-6 and ATF3, which indicate reduced inflammation and cell stress [64, 76, 77]. Whether these shifts in gene expression correlate to protein abundance or change in activity with a WD-fed mouse or LDLr -/- mice remains to be determined. With the observed cognitive impairment, and increase in BBB transport and neuroinflammation, we leveraged the collective and comprehensive expertise at our three universities to assess how a WD in LDLr-/- and WT mice shifts brain glucose uptake and metabolites.

A previous study by Hu *et al* showed changes in behavior were correlated to a decrease in brain glucose uptake in the thalamus and striatum of high-fat fed rodents [78]. Yet, our study showed an increase in ^18^FDG uptake in LDLr -/- mice. These disparities may in part be due to differences in the diet (higher % cholesterol and different source of fat), the rodent species (rat vs mouse), length of time spent on the diet (9 vs 12 weeks), or a difference in fasting vs fed state. Although observing perturbations in neurological activity in defined portions of the brain would be beneficial, current imaging technology has limited resolution (~1mm) and measurements in small regions are difficult.

While metabolic analysis of other cognitively impaired mouse models has shown elevation in lactate and glutamate [79, 80], we aimed to determine if hyperlipidemia alters metabolic pathways *in vivo* using ^1^H-MRS. Our ^1^H-MRS data indicated a significant increase in lactate levels with LDLr -/-, which has been observed previously in other cognitive decline models [79]. We also observed a trend for glutamine content to decrease.

To further assess metabolite disruption, we utilized GC/MS and LC/MS/MS to examine whether a broader range of pathways including: fatty acid metabolism, TCA cycle, and β-oxidation may contribute to the progression of cognitive decline. As anticipated, we detected a significant increase in C14:0 and C16:1 free fatty acids with diet, but the LDLr -/- genotype appeared to blunt the effects of diet on brain fatty acid and cholesterol profiles. It is feasible that genetic abolition of the LDL receptor (known as a contributing transporter of cargo into the brain [81]) could reduce transport of fatty acids into the brain despite elevated plasma lipid levels. Further evaluation of TCA cycle intermediates found an elevation in metabolic profile of aspartate, 2-hydroxygluterate, glutamine and lactate that appears to be predominately diet induced. This is interesting given that lactate, aspartate, and glutamate are increased in AD and db/db mouse models of cognitive decline [79, 80, 82] indicating that their elevation by WD may also be associated with the cognitive decline. While confounding, the difference in observed glutamate, lactate, and lipid moieties *in vivo* versus *ex vivo* may be due to methodology (GC/MS vs ^1^H-MRS), brain region (whole vs cortical focus), anesthesia interval effect (minutes vs hour), or genetic model (LDLr-/- vs db/db), yet, perturbations in lipid and metabolic intermediates are still present. Several studies have indicated that the type and depth of anesthesia can significantly modulate brain lactate and glutamine [83–85]. Additionally, the significant increase in GABA concentrations may suggest that GABA synthesis is induced by the WD in the LDLr-/- mutant mouse. Elevated cerebral spinal fluid levels of GABA have been linked to various neurological disorders including dementias, cerebellar cortical atrophy, and multiple sclerosis identifying this as a potential link between brain metabolic dysregulation and cognitive impairment [86].

WD in LDLr -/- mice showed decreased acyl-CoA species associated with fatty acid oxidation without altering the total acyl-CoA pool. These results suggest that there is a mismatch in fatty acid oxidation with diet or LDLr mutation suggestive of an inflammatory response due to oxidative stress-induced by altered lipid metabolism. These studies are consistent with previous studies that demonstrate hypometabolism precedes the cognitive decline of AD, where a decline in brain glucose metabolism and mitochondrial function can appear decades prior to diagnosis of AD [87]. Our studies show that LDLr may play a role in oxidative metabolism of lipids, as revealed by the changes in the short and medium chain acyl-CoA’s.

While we hypothesize that WD directly alters astrocyte-neuron metabolic balance, resulting in impaired cognitive function, elevated brain lactate and glutamine has also been associated with acute liver disease [88]. Hepatic encephalopathy was accompanied by elevated aspartate, glutamine, glucose, and lactate throughout the brain with increasing neuronal injury [89, 90]. While LDLr-/- mice had more severe liver disease than WT, it seems unlikely to have resulted in hepatic failure and it remains uncertain whether this would be sufficient to alter brain metabolism. Furthermore, the WD-fed LDLr -/- mouse model has predominately been used as a model for atherosclerosis and only more recently cognitive impairment [9, 91]. Regardless, these perturbations in systemic metabolism may lead to a derangement in neurotransmission and result in cognitive defects seen in these mouse models.

Interestingly, the altered cognitive function we see in the LDLr-/- are more modest than those seen in previous studies, which may be related in part to elevated phosphatidylcholine (PC) levels in the LDLr-/- mouse brain. The Cermenati *et al*. study showed that STZ diabetic mice had reduced myelin PC and may be linked to the neurodegenerative events in diabetes [92]. Further, we see an elevation in ceramide (d18:1/24:1), previously shown to have roles in arteriosclerosis, obesity, diabetes, and inflammation. In addition, long-chain sphingomyelins, derived from ceramides, activate macrophages inducing an inflammatory response [93]. These shifts in ceramides have been shown to have a role in astrocyte cell death and mediated cognitive impairment [94, 95]. However, the spatial distribution of lipid species is known to support the structural and metabolic functions of the central nervous system [96] and it remains to be determined if the spatial distribution of these metabolites are altered with WD.

Our oxylipins and endocannabinoids analysis demonstrated an increase in the 5-lipoxygenase (5-LOX) related metabolites including 5-HETE and Leukotriene LTB4. Elevated arachidonic acid (AA) and its 5-LOX products LTB4, LTD4, and 5-HETE have previously been shown to trigger apoptosis and suppress NFκB cell survival and are linked to hyperlipidemic inflammation [97, 98]. We also saw a decrease in anti-inflammatory 18:3n3 and 18:2n6 with a WD [99, 100]. However, this decrease may be due to a shift in dietary PUFA. The CD fat source is soybean oil, which provides 9.14g/kg of PUFAs while the WD, with milk fat provides 7.35g/kg (a decrease in n-3 PUFAS from 1.21 to 1.05 g/kg and n-6 PUFA’s from 7.93 to 6.3). Interestingly, in LDLr -/- mice, we saw an increase in the long chain (20 and 22 carbon) neuroprotective PUFAs [101], and a decrease in 9-Hydroxy-10,12-octadecadienoic acid (9-HODE), a pro-inflammatory mediator [102] and 13-HODE, shown to regulate platelet vessel wall adhesion [103, 104], and commonly associated with a protective effect. This may be due to a shift in the types or quantities of lipids normally delivered across the BBB [81].

It is of interest that while the LDLr-/- animals had the highest circulating lipid levels (cholesterol, triglycerides, HDL, and LDL), yet they did not show increased BBB transport, neuroinflammation, or cognitive impairment when compared to WT mice on WD. Having a constantly increased lipid level in the LDLr-/- mice may have a preconditioning/protective effect against the WD, or perhaps the temporal increase in circulating lipid levels associated with meals triggers metabolic and inflammatory pathways. Additionally, other undefined compensatory mechanism in the LDL-/- mice may protect the brain from elevated circulating lipids found in LDLr -/- mice. This indicates an increased serum lipid level alone may be insufficient to elicit these changes and that other factors may play a role. In contrast, studies have shown that high carbohydrate diets are sufficient to induce obesity, metabolic inflexibility, and inflammation [105, 106]. Further, increased dietary sucrose is sufficient to alter cognition [107–109] and altering the source of dietary carbohydrates (sucrose or cornstarch) has been shown to impact life span in rodent models [110]. While not significant, WT animals on a WD in our study, have the highest nominal insulin level, suggestive of a shift toward insulin resistance, a contributor to inflammation and cognitive impairment [111].

While we have focused on the metabolic, inflammatory and permeability changes associated with diet-induced altered cognitive function, others have hinted at the influence of reactive oxygen species (ROS) generated by a deregulated metabolism on cognitive decline. For instance, NADPH oxidase-derived production of ROS was shown to be involved in learning and memory impairments in 16-month-old female rats [112], macromolecular ROS damages neurons from aged WT and 3xTg-AD mice [113], and late stage AD patients show significant oxidative DNA damage [114]. Furthermore, in an older population, a Mediterranean diet rich in antioxidants is associated with improved cognitive function [115]. Future studies evaluating any shift in ROS and the antioxidant potential due to a Mediterranean diet remain to be evaluated as well as their influence on cognitive impairment [114].

In summary, we found that a WD shifts brain metabolism to a more stressed state profile, activates the inflammatory and vascular system in the brain, and increases BBB transport; all of which likely play a role in the observed alteration in cognitive function seen in WT and LDLr-/- mice. By better understanding how hyperlipidemia and insulin resistance influences neurovascular dysregulation we can better understand neurovascular inflammation-induced cognitive impairments and identify novel targets for the treatment of these debilitating disorders.
